# Ivosidenib enhances cisplatin sensitivity in ovarian cancer by reducing cancer cell stemness

**DOI:** 10.20517/cdr.2025.51

**Published:** 2025-04-24

**Authors:** Mengqing Chen, Lin Huang, Simei Zhao, Mengna Zhu, Si Sun, Wenhan Li, Jing Cai, Minggang Peng, Yiping Wen, Zehua Wang

**Affiliations:** Department of Obstetrics and Gynecology, Union Hospital, Tongji Medical College, Huazhong University of Science and Technology, Wuhan 430022, Hubei, China.; ^#^Authors contributed equally.

**Keywords:** Ivosidenib, cancer stem cells, platinum resistance, organoids

## Abstract

**Aim**: Cancer stem cells (CSCs) are pivotal in mediating platinum resistance in ovarian cancer. This study aimed to screen compounds sensitizing CSCs to cisplatin by using a small molecule inhibitor library.

**Methods**: A library of 105 common drugs was screened in ovarian CSC model SK-3rd and ovarian cancer platinum-resistant cell model SKDDP to identify those that could enhance sensitivity to cisplatin by MTT assay. The antitumor effect was assessed in ovarian cancer cells using the MTT assay, colony formation assay, and apoptosis assay. The impact on cancer cell stemness was evaluated using qPCR and Sphere-forming assays. Finally, the effect of the combination regimen was evaluated in patient-derived organoids (PDOs) under different treatments by the CellTiter-Glo Luminescence Assay.

**Results**: The results of the initial screening on SK-3rd identified five candidate compounds. Rescreening on SKDDP showed that Ivosidenib was the most effective in sensitizing cisplatin. MTT, colony formation, and apoptosis assays demonstrated that Ivosidenib enhanced the sensitivity to cisplatin, inhibited proliferation, and induced apoptosis in ovarian cancer cells, including SK-3rd and SKDDP. Furthermore, Ivosidenib lowered stemness marker expression and countered CSC enrichment caused by platinum-based chemotherapy in ovarian cancer cells. Finally, the synergistic effect of this combination was also confirmed in three ovarian cancer PDOs.

**Conclusion**: Ivosidenib may increase cisplatin sensitivity in ovarian cancer cells by decreasing their stemness, providing a potential therapeutic method for ovarian cancer patients.

## INTRODUCTION

Ovarian cancer remains the most lethal gynecologic malignancy, with a 5-year overall survival rate of less than 30%^[1]^. Due to the absence of effective early diagnostic strategies, most ovarian cancer cases are diagnosed at advanced stages. Currently, the standard treatment for ovarian cancer involves surgical resection combined with platinum-based chemotherapy^[2]^. While the majority of patients achieve an initial response to chemotherapy, approximately 70% develop recurrent disease, which frequently progresses to platinum resistance and ultimately results in therapeutic failure^[3]^. Given these challenges, enhancing platinum sensitivity and overcoming platinum resistance have emerged as critical priorities in the management of ovarian cancer.

Cancer stem cells (CSCs), alternatively termed tumor-initiating cells (TICs), represent a distinct subpopulation of malignant cells characterized by their capacity for self-renewal and differentiation^[4,5]^. Accumulating evidence suggests that CSCs are crucial in mediating chemotherapy resistance and promoting tumor recurrence^[6-8]^. Furthermore, several studies have demonstrated that platinum-based chemotherapy regimens can induce the enrichment of CSCs^[9-11]^. Consequently, targeting CSCs may offer a promising therapeutic approach to overcome platinum resistance in ovarian cancer.

In the domain of ovarian cancer treatment, despite the continuous emergence of novel pharmaceuticals, platinum-based drugs remain indispensable. Consequently, finding drugs to combine with platinum to enhance therapeutic efficacy has been actively pursued. With the development of modern screening techniques, including small molecule inhibitor screening, siRNA library screening, and CRISPR-Cas9 library screening, many drug combination regimens have been identified for cancer therapy. For instance, through the integration of small-molecule inhibitor screening and CRISPR-Cas9 library screening, researchers have demonstrated that GSK-J4 and donafenib exhibit significant synergistic effects in hepatocellular carcinoma^[12]^. CRISPR-Cas9 high-throughput screening has identified theaflavin, a bioactive component derived from tea, as a compound that overcomes multidrug resistance by inducing apoptosis, offering a promising therapeutic strategy for refractory ovarian cancer^[13]^. Based on these findings, we employed a small molecule compound library to screen for compounds capable of enhancing the platinum sensitivity of ovarian CSCs. The results showed that Ivosidenib, an Isocitrate Dehydrogenase 1 (IDH1) inhibitor, significantly enhanced cisplatin sensitivity in both the CSCs and cisplatin-resistance models.

Ivosidenib is a selective inhibitor of mutant IDH1, which effectively reduces the production of the carcinogen 2-Hydroxyglutarate (2-HG)^[14]^. In relapsed or refractory Acute Myeloid Leukemia (AML), Ivosidenib has demonstrated clinical efficacy as a monotherapy, and further clinical trials are ongoing for other cancers^[15-17]^. However, no relevant studies have been conducted on ovarian cancer.

In this study, we demonstrated that Ivosidenib significantly enhances cisplatin sensitivity and suppresses cancer stemness in ovarian cancer cells through a series of *in vitro* assays, including colony formation, apoptosis analysis, qPCR, and Aldefluor assays. These findings were further validated in patient-derived organoid (PDO) models of ovarian cancer, providing a potential therapeutic method for ovarian cancer patients.

## METHODS

### Cell lines and culture

Ovarian cancer cell lines, including SKOV3, CAOV3, and OVCAR4, were obtained from the China Center for Type Culture Collection (CCTCC) at Wuhan University. All cell lines were cultured in DMEM/F12 (L310KJ, BasalMedia, Shanghai, China) supplemented with 10% fetal bovine serum (BC-SE-FBS01, Biochannel, Nanjing, China) and 1% penicillin-streptomycin (S110JV, BasalMedia). Cells were maintained at 37 °C in a humidified incubator with 5% CO_2_. The medium was refreshed every 2-3 days, and cells were subcultured upon reaching 80%-90% confluence using 0.25% trypsin-EDTA (S310KJ, BasalMedia). To ensure the absence of mycoplasma contamination, all cell lines were routinely tested using the MycoBlue Mycoplasma Detector (D101, Vazyme, Nanjing, China) according to the manufacturer’s protocol.

### Agents

Ivosidenib (an IDH1 inhibitor, 1448347-49-6, MedChemExpress, Monmouth Junction, NJ, USA) was dissolved in dimethyl sulfoxide (DMSO), and cisplatin (S1166, Selleck, Houston, TX, USA) was dissolved in ddH_2_O. Both compounds were aliquoted and stored at -20 °C for long-term use. *In vitro*, DMSO was present at a concentration of less than 0.01% (v/v) to avoid potential solvent toxicity.

### Clinical samples

Human ovarian cancer samples were used under the Declaration of Helsinki, with approval from the Research Ethics Committee of Union Hospital, Tongji Medical College, Huazhong University of Science and Technology (Wuhan, China). Informed consent was obtained from all patients. All processes were performed following the university's scientific research standards, and samples were delivered to the lab within half an hour.

### Human ovarian cancer organoid culture

Human ovarian cancer tissues obtained in the operating room were mechanically minced into 1 mm^3^ pieces and digested in 2 mg/mL collagenase (BS164, Biosharp, Hefei, Anhui, China) prepared in basal medium for 1 h at 37 °C. After filtering through a 40 μm cell filter, the cell suspension was centrifuged at 430 g for 5 min. The cell pellet was washed with HBSS containing 2% FBS and 1% HEPES, followed by red blood cell lysis and additional washing to isolate single human ovarian cancer cells. Ovarian cancer cells were mixed with Matrigel, seeded at 10 µL per drop in preheated six-well plates, solidified at 37 °C for 30 min, and cultured in organoid medium. Organoid culture medium: DMEM/F12 supplemented with 1% penicillin-streptomycin, 1 × Glutamax (35050061, Gibco), 1% HEPES (07200, Stemcell), 1 × B27 (05731, Stemcell), 100 ng/mL Noggin (120-10C, Peprotech), 50 ng/mL EGF (AF-100-15, Peprotech), 100 ng/mL R-spondin 1 (120-38, Peprotech), 10 ng/mL FGF-10 (10026, Peprotech), 10 ng/mL FGF2 (100-18B, Peprotech), 0.02 μg/mL Wnt3a (5036-WN, R&D systems), 0.05 μg/mL human NRG1 (5898-NR, R&D systems), 10 mM nicotinamide (N0636, Sigma Aldrich), 1.25 mM N-acetylcysteine (A9165, Sigma Aldrich), 10 nM 17-β-Estradiol (2824, R&D systems), 10 μM SB202190 (S7076, Sigma Aldrich), 500 nM A83-01 (SML0788, Sigma Aldrich), and Y-27632 dihydrochloride (72302, Stemcell). For drug treatment, the working concentrations of these drugs were as follows: 20 μM cisplatin (S1166, Selleck) and 10 μM Ivosidenib (1448347-49-6, MedChemExpress). The medium was changed every 3-4 days. Passages were made every 1-3 weeks, depending on the density and size of the organoids.

### CellTiter-Glo Luminescence Assay

Organoid viability was assessed using the CellTiter-Glo® 3D Cell Viability Assay (G9681, Promega) according to the manufacturer’s instructions. Briefly, the culture medium was removed, and each well was supplemented with 100 μL of pre-warmed detection reagent. After incubation at room temperature for 10 min to ensure complete organoid lysis, luminescence intensity was measured using a Varioskan™ LUX multimode microplate reader (Thermo Fisher Scientific).

### MTT assay

Cytotoxicity was assessed using the MTT assay. Cells were seeded at a density of 5,000 cells per well in 96-well plates and cultured in 200 µL of DMEM/F12 medium supplemented with 10% fetal bovine serum for 24 h at 37 °C. After treatment with the indicated compounds for 72 h, the medium was carefully aspirated, and 200 µL of fresh medium containing 10% MTT (3-(4,5-dimethylthiazol-2-yl)-2,5-diphenyltetrazolium bromide) was added. The plates were then incubated for 4 h at 37°C. Following incubation, the supernatant was carefully removed, and 100 µL DMSO was added to each well to solubilize the formazan crystals. The plates were incubated at room temperature for 10 min, and the absorbance was measured at 570 nm using a microplate reader. The values of half-maximal inhibitory concentration (IC_50_) were calculated using GraphPad Prism 9.5.0 software.

### Colony formation assay

Ovarian cancer cells were seeded at a density of 500 cells per well in six-well plates. After cell attachment, cells were treated with 10 µM cisplatin, 10 µM Ivosidenib alone, or a combination of both, and cultured in a humidified incubator at 37 °C with 5% CO_2_ until visible colonies formed. Subsequently, the cells were fixed with 4% paraformaldehyde for 30 min at room temperature, followed by staining with 0.1% crystal violet for at least 1 h. Colonies consisting of more than 50 cells were manually counted and included in the analysis. The experiment was performed in triplicate using independent biological replicates.

### RNA extraction and qRT-PCR

Total cellular mRNA was extracted using an automated nucleic acid extractor (Vazme, VNP-32P, Nanjing, China). Reverse transcription was performed using the 5 × Hifair® Ⅲ Reverse Transcriptase Buffer (15662ES, Yeasen Shanghai, China). 2× HieffCanace™PCR Master Mix (10138ES, Yeasen)was used for real-time fluorescence quantitative PCR (qRT-CR) on the qTOWER3 real-time PCR system (Analytik Jena). *ACTB* was used as the internal control, and relative mRNA expression was calculated as 2^−ΔΔCt^. The primer sequences are shown as follows: *ACTB*: forward 5’-GCCAACACAGTGCTGTCTGG-3’, reverse 5’-GCTCAGG AGGAGCAATGATCTTG-3’; *ALDH1A1*: forward 5’-CCACTCACT GAATCATG CCA-3’, reverse 5’-TGAGCCAGTCACCTGTGTTC-3’; *CD44*: forward 5’-CT GCCGCTTTGCAGGTGTA-3’, reverse 5’-CATTGTG GGCAAGGTGCTATT-3’; *BMI-1*: forward 5’-CCACCTGATGTGTGTGCTTTG-3’, reverse 5’-TTCAGTAG TGGTCTGGTCTTGT-3’; *KLF-4*: forward 5’-CAGCTTCACCTATCCGATCCG-3’, reverse 5’-GACTCCCTGCCATAGAGGAGG-3’.

### Apoptosis analysis

Ovarian cancer cells were seeded in six-well plates and treated with 10 µM cisplatin, 10 µM Ivosidenib alone, or a combination of both at 40-50% cell confluence. After 72 h of treatment, apoptosis was assessed using the Annexin V-FITC/PI Apoptosis Detection Kit (A211, Vazyme) according to the manufacturer’s instructions. Flow cytometry analysis was performed using the ID7000 Spectral Cell Analyzer (Sony Biotechnology, USA). Data analysis was performed using FlowJo 10.8.1 software. Annexin V+/PI− cells were defined as early apoptotic cells, while Annexin V+/PI+ cells were classified as late apoptotic cells. The total apoptosis rate was calculated as the percentage of the sum of early and late apoptotic cell populations relative to the total cell count. Each experiment was conducted with three independent biological replicates.

### Aldefluor assay

The ALDH1 activity in SKOV3, CAOV3, OVCAR4, SKDDP, and SK-3rd cells was assessed following treatment with 10 µM cisplatin, 10 µM Ivosidenib, or their combination, using the Aldefluor Assay Kit (01700, Stemcell Technologies, Edmonton, Alberta, Canada) according to the manufacturer's instructions. ALDH1 activity was quantified using the ID7000 spectral cell analyzer. Data analysis was performed using FlowJo 10.8.1, with ALDH1+ cells identified based on fluorescence intensity relative to the DEAB-treated control. Each experiment was conducted in three independent biological replicates.

### Sphere-forming assay

A total of 10³ cells were seeded on ultra-low adhesion plates (Corning, NY, US) in 2 mL of stem cell medium and treated with either DMSO or 10 µM Ivosidenib. The stem cell culture medium consisted of serum-free DMEM/F12 supplemented with 2% B-27 supplement without vitamin A (Invitrogen, Carlsbad, CA, USA), 20 ng/mL basic fibroblast growth factor (bFGF, Peprotech, USA), 20 ng/mL epidermal growth factor (EGF, Peprotech, USA), 10 ng/mL leukemia inhibitory factor (LIF, Peprotech, USA), and insulin-transferrin-selenium (ITS, Invitrogen, USA). Sphere formation was monitored daily, and tumor spheres larger than 50 µm in diameter were photographed and counted after 14 days. Statistical analysis of sphere formation was conducted using GraphPad Prism 9.5.0, with three independent biological replicates for each experiment.

### SKDDP and SK-3rd models

The cisplatin-resistant cell line we used, SKDDP, was a subline of the SKOV3 cell line. We established the cisplatin-resistant subline by gradually exposing SKOV3 cells to multicycles of cisplatin treatment with increasing concentrations *in vitro*. First, the IC_50_ of SKOV3 parental cells treated with cisplatin was determined using the MTT assay. Cells in the logarithmic growth phase were selected and treated with a drug concentration equivalent to 10% of the IC_50_. After reaching 50% cell density, the medium was changed to a drug-free medium for further culture. At 80% density, cells were passaged and the above procedures were repeated 6-8 times with gradual increases in drug concentration. The IC_50_ of drug-resistant cells was determined, and the drug resistance index (RI) was calculated. An RI > 5 indicated successful construction of the resistant cell line.

To establish the SK-3rd stem cell model, the human ovarian cancer cell line SKOV3 was subcutaneously injected into the right scapular region of 4-week-old female BALB/c nude mice (Vital River, Beijing, China) at a density of 1 × 10^6^ cells per mouse. When the subcutaneous tumor reached approximately 0.5 cm in diameter, the mice were treated with cisplatin (3 mg/kg/day, i.t.). When the tumor reached 1.5 cm in diameter, it was enzymatically digested with collagenase to generate a single-cell suspension. The obtained tumor cells were cultured in suspension. After two weeks, the tumor cells were harvested and transplanted into female BALB/c nude mice as previously described. The first, second, and third generations of cisplatin-treated xenografted tumor cells were designated as SK-1st, SK-2nd, and SK-3rd, respectively.

### Small molecule compound screening

A customized library consisting of 105 compounds purchased from Selleck Chemicals was used for the screening. SK-3rd cells were seeded at 5000 cells per well in 96-well plates. The following day, the 96-well plates were divided into two groups: one group was treated with 105 small-molecule compounds, while the other group was treated with a combination of cisplatin and small-molecule compounds (*n* = 4). After 72 h of treatment, cell viability was assessed using the MTT assay according to the manufacturer’s instructions.

Compounds meeting the following criteria were selected for further analysis: (1) SK-3rd cell viability > 90% when treated with the small-molecule compound alone; and (2) SK-3rd cell viability <60% when treated with the small-molecule compound in combination with cisplatin. Subsequently, the initially screened compounds were further evaluated in the ovarian cancer cisplatin-resistant cell model SKDDP to determine the best cisplatin-sensitizing compounds.

**Figure 1:**
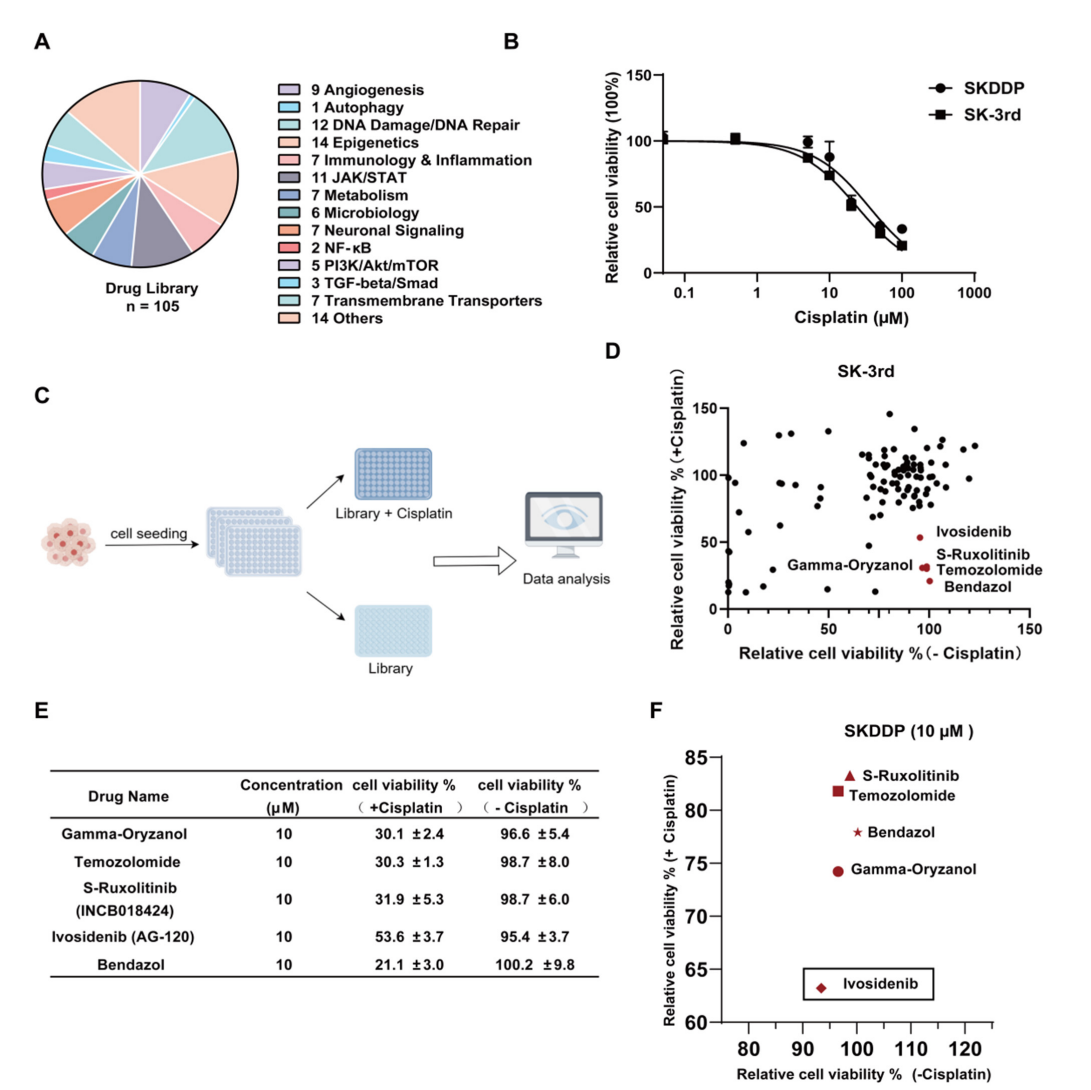
Screening of a small molecule compound library shows that Ivosidenib enhances the sensitivity of ovarian cancer cells to cisplatin. (A) Breakdown of the compound library based on the molecular pathways associated with the compound targets. (B) Dose-response curves for SK-3rd and SKDDP treated with cisplatin for 72 h. (C) Schematic of the compound screening.(D) Scatter plot showing cell viability in SK-3rd cells treated with compound library alone or the compound library + cisplatin. The red dots represent agents that resulted in over 90% survival of SK-3rd cells alone but less than 60% survival when combined with cisplatin (*n* = 4). (E) Corresponding compound names, concentrations, and cell viability data of compounds with > 90% cell viability when used alone but < 60% cell viability combined with 10 µM cisplatin.(F) Scatter plot showing cell viability in SKDDP cells treated with the five selected compounds alone or with the compounds plus cisplatin at a concentration of 10 µM (*n* = 4).

We used a working concentration of 10 µM for all compounds. This information has been detailed in Figure 1A and Supplementary Table 1. The concentration of cisplatin was set at 10 µM, corresponding to the IC_20_ value for SK-3rd cells.

### Statistical analyses

GraphPad Prism 9.5.0 was used to present and analyze all data. Data are expressed as the mean ± SD (*n* ≥ 3). Comparisons between the two groups were performed using an unpaired Student's t-test. Comparisons among three or more groups were performed using one-way analysis of variance (ANOVA) followed by Tukey's post hoc test for multiple comparisons. The effects of two factors on experimental outcomes were analyzed using a two-way analysis of variance (ANOVA) followed by Tukey's post hoc test for multiple comparisons. Statistical significance levels were indicated as ns (not significant), *P < 0.05, **P < 0.01, ***P < 0.001, and ****P < 0.0001.

## RESULTS

### Screening of a small molecule compound library shows that Ivosidenib enhances the sensitivity of ovarian cancer cells to cisplatin

To search for compounds that could increase the sensitivity of ovarian cancer cells to cisplatin, we purchased a library of 105 small molecule compounds from Selleck, which targets diverse cellular signaling pathways, including apoptosis, DNA damage and repair, epigenetic regulation, autophagy, and other critical biological processes [Figure 1A and Supplementary Table 1]. The screening was performed using SK-3rd cells, an ovarian CSC model that had been successfully established in a previous laboratory study^[18]^. We assessed the stemness of SK-3rd cells versus parent SKOV3 cells by using the Aldefluor assay. The results showed that the proportion of ALDH1+ cells in SK-3rd was significantly higher than that in SKOV3 cells (30.1% *vs.* 5.06%, *P* < 0.001), confirming the successful construction of the stem cell model [Supplementary Figure 1A-C]. Based on the approximate IC_20_ of SK-3rd cells, we used 10µM cisplatin for library screening, and all compounds in the library were at a working concentration of 10 µM [Figure 1B and C]. Using a screening logic where compounds with relative cell viability > 90% alone and < 60% in combination with cisplatin were prioritized, we identified five compounds, including Gamma-Oryzanol, Temozolomide, S-Ruxolitinib, Ivosidenib, and Bendazol [Figure 1D and E].

To further screen for compounds that sensitize cisplatin in ovarian cancer, we constructed a cisplatin-resistant cell model, SKDDP, derived from SKOV3 cells. The drug resistance index of SKDDP surpassed 5, as determined by the MTT assay, confirming the successful development of a cisplatin-resistant cell line [Supplementary Figure 1D and E]. Subsequently, secondary screening was conducted in SKDDP cells. The MTT assay was employed to evaluate the cytotoxic effects of the five candidate drugs at concentrations of 1 μM, 5 μM, 10 μM, 20 μM, and 50 μM, either alone or in combination with cisplatin. The results indicated that Ivosidenib exerted the strongest sensitizing effect on cisplatin at 1-10 μM. Intriguingly, at higher concentrations (20-50 μM), the other candidate drugs not only failed to enhance cisplatin cytotoxicity but also exhibited an antagonistic effect, wherein the combination treatment resulted in reduced cell killing compared to single-agent therapy [Figure 1F and Supplementary Figure 1F]. Consequently, we selected Ivosidenib for further investigation, as it is the only drug that demonstrated a sensitizing effect on cisplatin in both cell models, despite its less pronounced performance in SK-3rd.

### Ivosidenib increases the sensitivity of ovarian cancer cells to cisplatin

**Figure 2:**
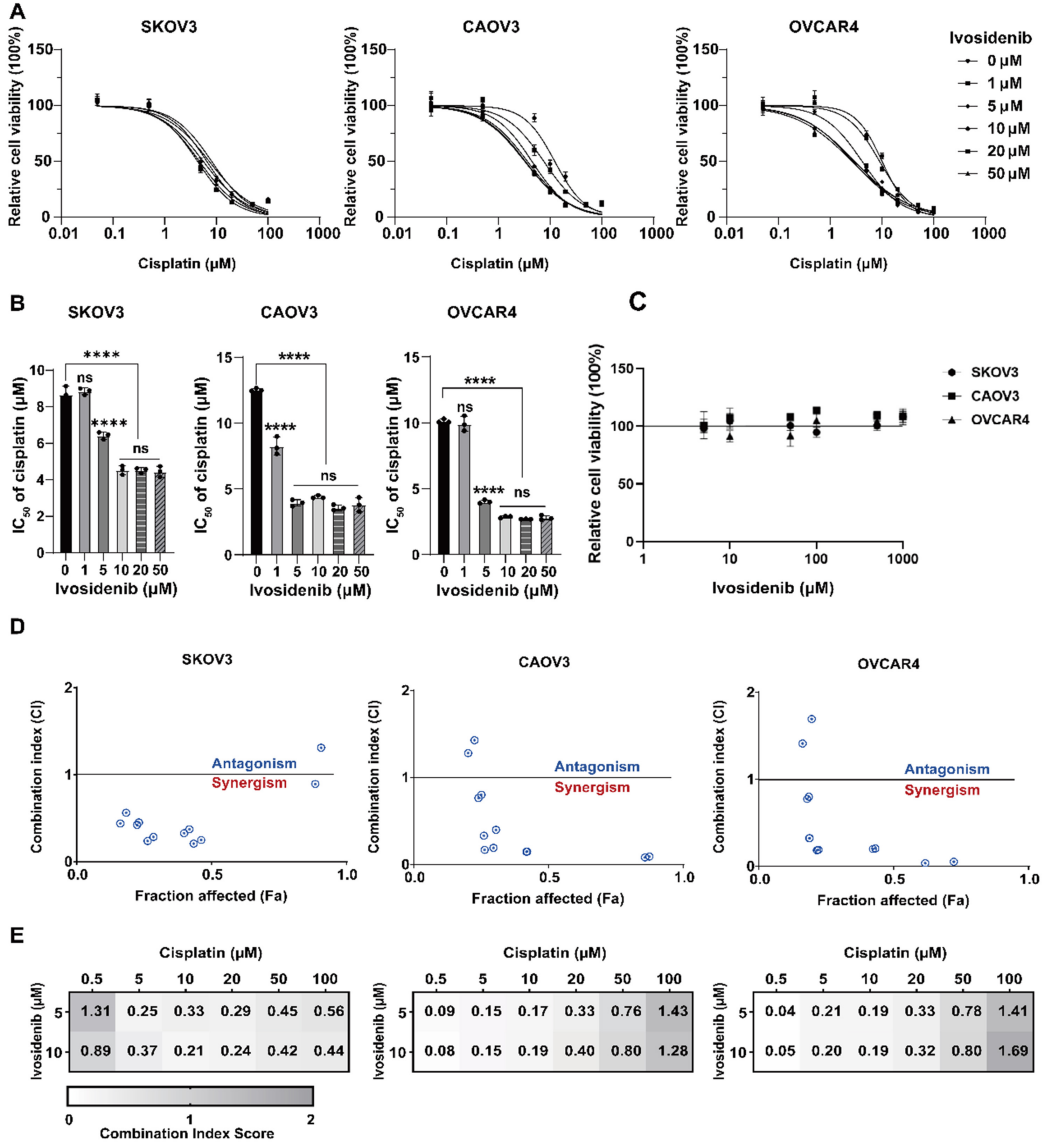
Ivosidenib increases the sensitivity of ovarian cancer cells to cisplatin. (A) Dose-response curves of SKOV3, CAOV3, and OVCAR4 detected by MTT assay treated with cisplatin and 0 μM, 1 μM, 5 μM, 10 μM, 20 μM, and 50 μM Ivosidenib for 72 h. Data represent mean ± SD of three biologically independent experiments. (B) The lC_50_ of SKOV3, CAOV3, and OVCAR4 to cisplatin in (A) were calculated by Graph Pad Prism 9.5.0. Data represent mean ± SD of three biologically independent experiments. ns, not significant, ***P < 0.001; ****P < 0.0001 using a one-way ANOVA followed by Tukey's post hoc test for multiple comparisons. (C) Dose-response curves of SKOV3, CAOV3, and OVCAR4 were detected by the MTT assay and treated with Ivosidenib after 72 h. Data represent mean ± SD of three biologically independent experiments. (D) Fa-CI plot of combined treatments of cisplatin (0.5 μM, 5 μM, 10 μM, 20 μM, 50 μM, 100 μM) and Ivosidenib (5 μM, 10 μM) of SKOV3, CAOV3, and OVCAR4 cells. The combination index was calculated using CompuSyn software. CI < 1, =1, and >1 indicate synergistic, additive, and antagonistic effects, respectively. CI, Combination index. Fa, Fraction affected. Each score represents data from three biologically independent experiments. (E) CI scores of SKOV3, CAOV3, and OVCAR4 cells treated with Ivosidenib in combination with cisplatin at the indicated concentrations. Each CI score represents data from three biologically independent experiments.

We detected the IC_50_ values of cisplatin and carboplatin for all ovarian cancer cell lines in our laboratory and selected three relatively resistant parental cell lines, SKOV3, CAOV3, and OVCAR4, for subsequent experiments [Supplementary Figure 2A and B]. To further validate the screening results, we first evaluated the effect of 1 μM, 5 μM, 10 μM, 20 μM, and 50 μM Ivosidenib on the cisplatin sensitivity of these cell lines using an MTT assay. The results showed that Ivosidenib sensitizes ovarian cancer cells to cisplatin, as evidenced by a decreased IC_50_. This sensitization exhibited concentration dependence at low doses (< 10 μM) but reached a plateau at concentrations exceeding 10 μM [Figure 2A and 2B]. When SKOV3, CAOV3, and OVCAR4 were treated with Ivosidenib monotherapy, cell viability was almost unaffected [Figure 2C].

To further investigate whether Ivosidenib could sensitize ovarian cancer cells to carboplatin, another commonly used platinum-based agent, we performed MTT assays using varying concentrations of Ivosidenib and carboplatin. In contrast to the observed effects with cisplatin, Ivosidenib did not enhance the cytotoxicity of carboplatin in ovarian cancer cells [Supplementary Figure 2C and D]. These results suggest that Ivosidenib does not universally sensitize ovarian cancer cells to all platinum-based drugs but exhibits a specific sensitizing effect on certain agents, such as cisplatin.

The combination index (CI) is a quantitative metric used to evaluate the effects of drug combinations. CI < 1 indicates a synergistic interaction between the drugs, CI = 1 suggests an additive effect, and CI > 1 implies an antagonistic interaction between the drugs^[19]^. To explore the synergistic effects of cisplatin in combination with Ivosidenib, we used CompuSyn software to calculate CI values and growth inhibition of cells treated with Ivosidenib administered at concentrations of 5 µM and 10 µM, and cisplatin at concentrations of 5 µM, 10 µM, 20 µM, and 50 µM. The results revealed that in SKOV3, CAOV3, and OVCAR4 cells, the combined treatment of cisplatin and Ivosidenib showed CI < 1 at some concentration combinations, suggesting a synergistic antitumor activity *in vitro* [Figure 2D and E].

### Ivosidenib combined with cisplatin reduces colony formation and enhances apoptosis in ovarian cancer cells

**Figure 3:**
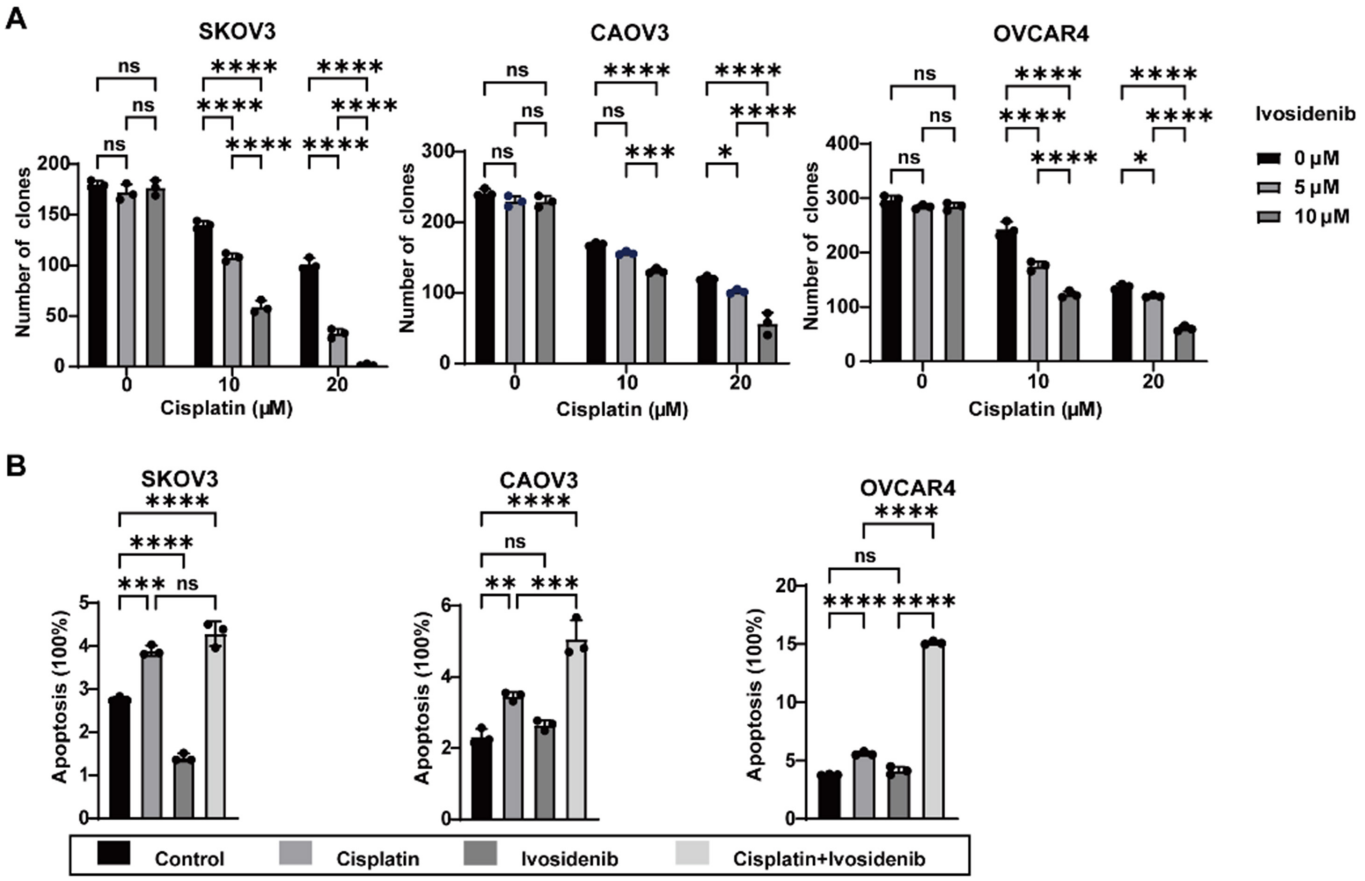
Ivosidenib combined with cisplatin reduces colony formation and enhances apoptosis in ovarian cancer cells. (A) Quantification of cell colony number in SKOV3, CAOV3, and OVCAR4 cells treated with Ivosidenib, cisplatin, or their combination. Data represent mean ± SD of three biologically. ns, not significant, *P < 0.05; ***P < 0.001; ****P < 0.0001 using a two-way ANOVA followed by Tukey's post hoc test for multiple comparisons. (B) Quantification of cell apoptosis analysis in SKOV3, CAOV3, and OVCAR4 cells treated with 10 μM Ivosidenib, 10 μM cisplatin, or their combination for 72 h. Data represent mean ± SD of three biologically. ns, not significant, **P < 0.01; ***P < 0.001; ****P < 0.0001 using a one-way ANOVA followed by Tukey's post hoc test for multiple comparisons.

We conducted colony formation assays to systematically evaluate the effects of Ivosidenib and cisplatin, both individually and in combination, on the proliferative capacity of SKOV3, CAOV3, and OVCAR4. The results indicated that Ivosidenib alone had minimal influence on the number of cell colonies, while cisplatin alone partially reduced colony formation. In contrast, the combination treatment significantly reduced the number of cell colonies compared to monotherapy [Figure 3A and Supplementary Figure 3A].

In addition, apoptosis assays were conducted to determine whether the combination of cisplatin and Ivosidenib induces greater levels of apoptosis. The results demonstrated that Ivosidenib alone did not significantly induce apoptosis but enhanced the apoptotic effects induced by cisplatin [Figure 3B and Supplementary Figure 3B].

### Ivosidenib increases the sensitivity of SKDDP and SK-3rd to cisplatin

**Figure 4:**
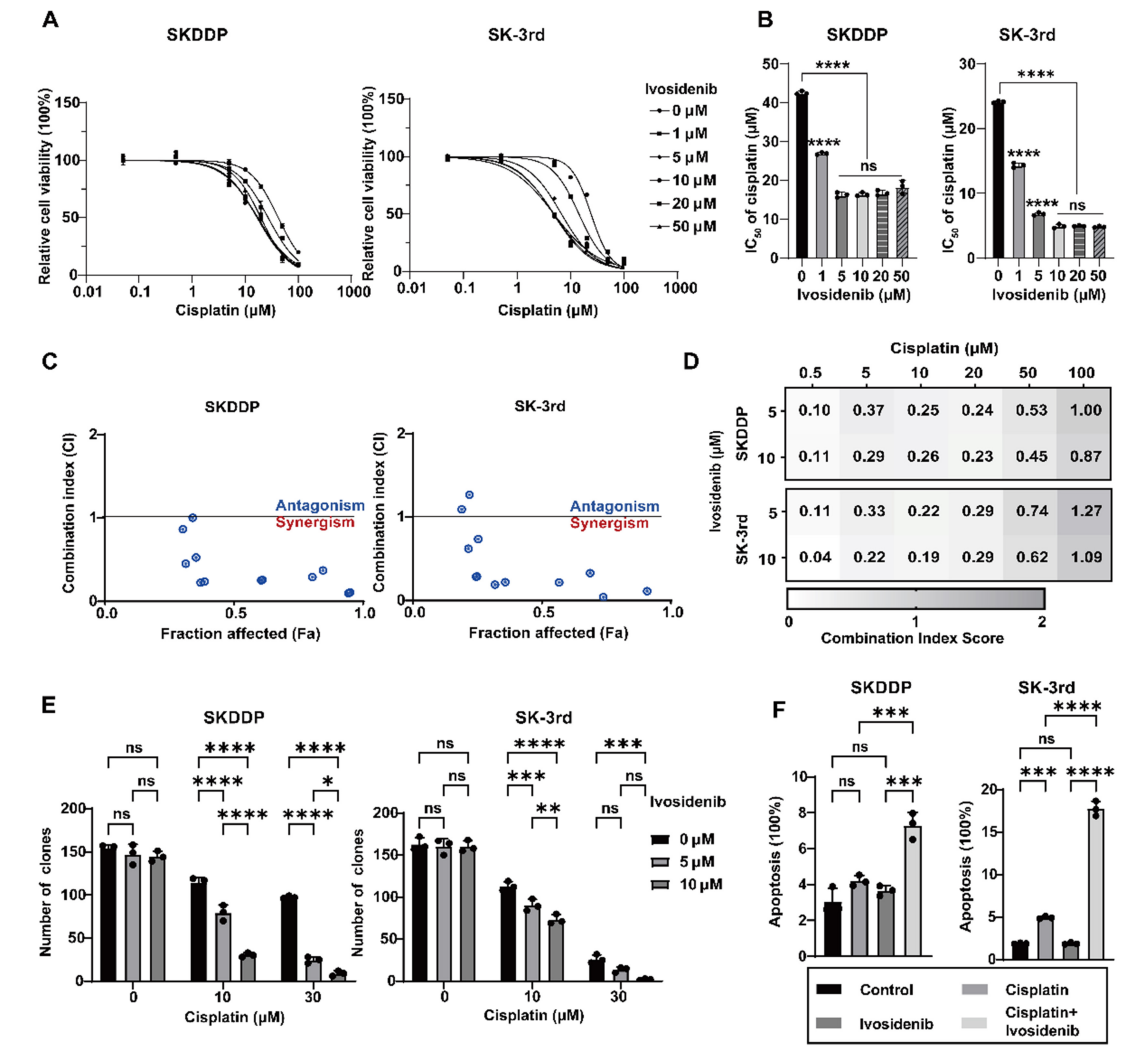
Ivosidenib increases the sensitivity of SKDDP and SK-3rd to cisplatin. (A) Dose-response curves of SKDDP and SK-3rd detected by MTT assay treated with cisplatin and 0 μM, 1 μM, 5 μM, 10 μM, 20 μM, and 50 μM Ivosidenib for 72 h. Data represent mean ± SD of three biologically independent experiments. (B) The lC_50_ of SKDDP and SK-3rd to cisplatin in (A). Data represent mean ± SD of three biologically independent experiments. ns, not significant, *P <0.05; **P < 0.01; ***P < 0.001; ****P < 0.0001 using a one-way ANOVA followed by Tukey's post hoc test for multiple comparisons. (C) Fa-CI plot of combined treatments of cisplatin (0.5 μM, 5 μM, 10 μM, 20 μM, 50 μM, 100 μM) and Ivosidenib (5 μM, 10 μM) of SKDDP, and SK-3rd cells. The combination index was calculated using CompuSyn software. CI < 1, =1, and >1 indicate synergistic, additive, and antagonistic effects, respectively. CI, Combination index. Fa, Fraction affected. Each score represents data from three biologically independent experiments. (D) CI scores of SKDDP and SK-3rd cells treated with Ivosidenib in combination with cisplatin at the indicated concentrations. Each CI score represents data from three biologically independent experiments. (E) Quantification of cell colony number in SKDDP and SK-3rd cells treated with Ivosidenib, cisplatin, or their combination for 72 h. Data represent mean ± SD of three biologically independent experiments. ns, not significant, *P <0.05; **P < 0.01; ***P < 0.001; ****P < 0.0001 using a two-way ANOVA followed by Tukey's post hoc test for multiple comparisons. (F) Quantification of cell apoptosis analysis in SKDDP and SK-3rd cells treated with DMSO, 10 μM Ivosidenib, 10 μM cisplatin, or their combination for 72 h. Annexin V-positive cells were analyzed by flow cytometry after treatment. Data represent mean ± SD of three biologically independent experiments. ns, not significant, ***P < 0.001; ****P < 0.0001 using a one-way ANOVA followed by Tukey's post hoc test for multiple comparisons.

To investigate the sensitizing effect of Ivosidenib on ovarian cancer cisplatin-resistant cells and CSCs, we conducted a series of experiments using the established SKDDP and SK-3rd cell models. The MTT assay results demonstrated that Ivosidenib similarly enhanced cisplatin sensitivity in both SKDDP and SK-3rd cells, mirroring the effects observed in parental cell lines [Figure 4A and B]. Subsequently, we employed CompuSyn software to evaluate potential synergistic effects between Ivosidenib (5 µM and 10 µM) and cisplatin (0.5-100 µM) at various concentration combinations. The analysis revealed CI values < 1 across multiple dose combinations, indicating a synergistic interaction between cisplatin and Ivosidenib [Figure 4C and D].

Consistent with the findings in parental ovarian cancer cells, colony formation and apoptosis assays showed that Ivosidenib alone did not significantly affect the proliferation or apoptosis of SKDDP and SK-3rd cells. However, the combination of Ivosidenib and cisplatin significantly reduced colony formation and increased apoptosis rates in SKDDP and SK-3rd cells [Figure 4E and F, Supplementary Figure 4A and B]. Taken together, these results indicate that Ivosidenib exerts synergistic effects in ovarian cancer cisplatin-resistant cells and CSCs, inhibiting cell proliferation and promoting apoptosis.

### Ivosidenib reduces the stemness of ovarian cancer cells

**Figure 5:**
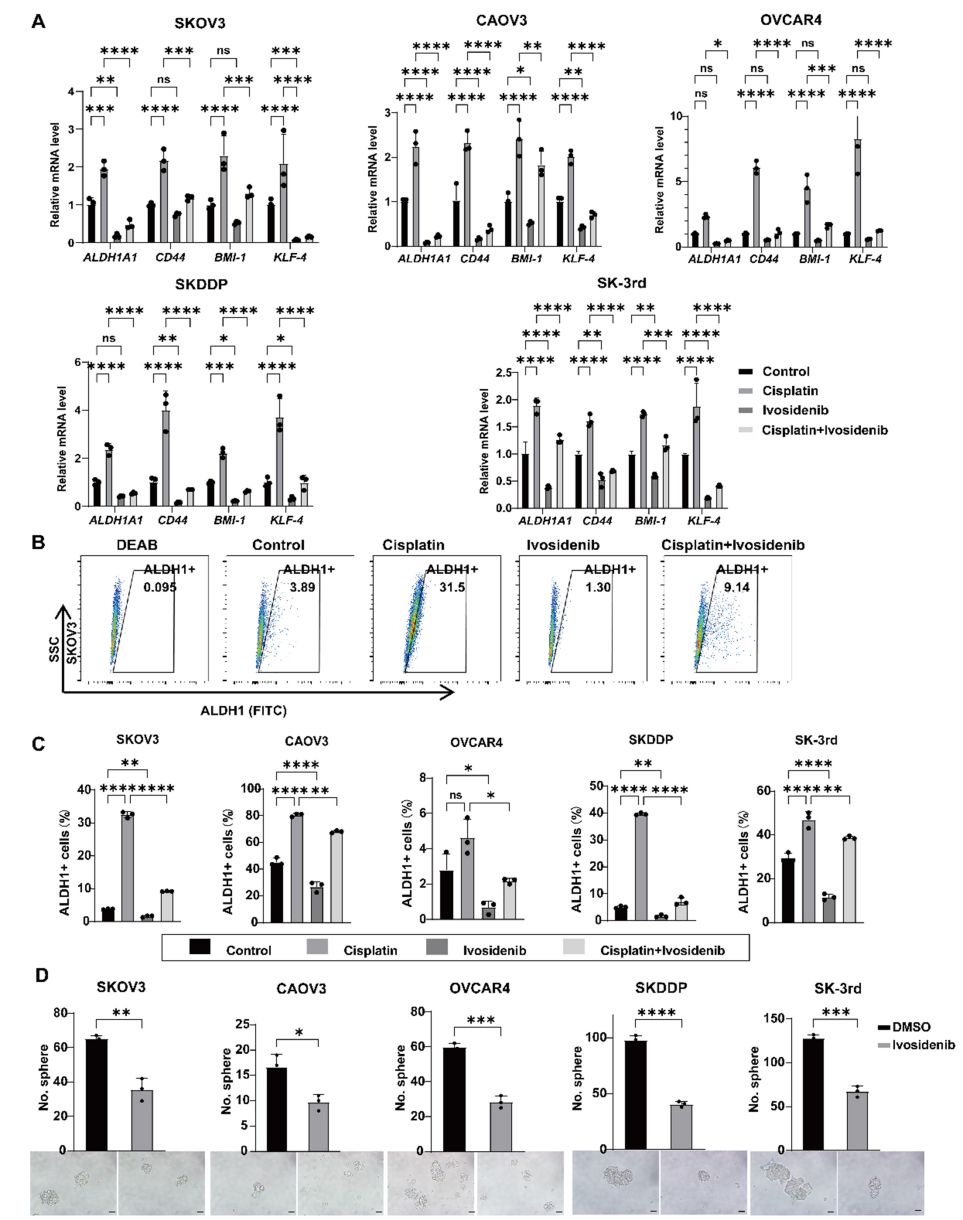
Ivosidenib reduces the stemness of ovarian cancer cells. (A) Quantitative analysis of the mRNA levels of stemness-related genes, including *ALDH1A1*, *CD44*, *BIM-1*, and *KLF-4* in SKOV3, CAOV3, OVCAR4, SKDDP, and SK-3rd treated with DMSO, 10 μM Ivosidenib, 10 μM cisplatin, or their combination for 72 h by qRT-PCR. Data are represented as mean ± SD of three biologically independent experiments. ns, not significant, *P <0.05; **P < 0.01; ***P < 0.001; ****P < 0.0001 using a one-way ANOVA followed by Tukey's post hoc test for multiple comparisons. (B) Representative images of ALDH1+ cells were analyzed using flow cytometry in SKOV3 cells treated with DMSO, 10 μM Ivosidenib, 10 μM cisplatin, or their combination for 72 h. DEAB was used as the negative control for ALDH1 activity. (C) The proportion of ALDH1+ cells was analyzed statistically in SKOV3, CAOV3, OVCAR4, SKDDP, and SK-3rd. Data are represented as mean ± SD of three biologically independent experiments. ns, not significant, *P <0.05; **P < 0.01; ***P < 0.001; ****P < 0.0001 using a one-way ANOVA followed by Tukey's post hoc test for multiple comparisons. (D) Representative images and statistical histograms of spheres generated by SKOV3, CAOV3, OVCAR4, SKDDP, and SK-3rd cells treated with DMSO or 10 μM Ivosidenib. Scale bar: 50 μm. *P <0.05; **P < 0.01; ***P < 0.001; ****P < 0.0001 using an unpaired Student's *t*-test.

Given that therapy resistance in tumors has been linked to CSCs, we investigated whether Ivosidenib could reduce the stemness characteristics of ovarian cancer cells. To achieve this, we utilized qPCR to evaluate the mRNA expression levels of stemness-related genes *(ALDH1A1*, *CD44*, *BMI-1*, *KLF-4*, *SOX2*, *OCT4*, and *NANOG*) in the ovarian cancer cell lines SKOV3, CAOV3, OVCAR4, SK-3rd, and SKDDP treated with cisplatin and Ivosidenib either individually or in combination. The results indicated that cisplatin significantly increased the mRNA expression levels of these stemness-related genes. In contrast, Ivosidenib effectively decreased the expression of stemness markers and partially mitigated the cisplatin-induced upregulation of stemness-related genes [Figure 5A and Supplementary Figure 5A].

To further assess the impact of Ivosidenib on ovarian cancer stemness, we performed Aldefluor assays. Consistent with the previous results, cisplatin significantly increased ALDH1 activity in ovarian cancer cells compared to the control group. Conversely, Ivosidenib reduced ALDH1 activity and counteracted the cisplatin-induced increase in ALDH1 activity in ovarian cancer cells [Figure 5B and C, Supplementary Figure 5B]. Additionally, we assessed the effect of Ivosidenib on the protein expression of stemness markers ALDH1A1 and SOX2 by Western blot. The results demonstrated that treatment with 10 μM Ivosidenib reduced the expression of ALDH1A1 and SOX2 in ovarian cancer cells compared to the DMSO control group [Supplementary Figure 5C]. CSCs possess a self-renewal capacity, and tumorsphere formation indicates this ability^[20]^. Therefore, we performed the sphere-forming assay to evaluate the effect of Ivosidenib on ovarian cancer cell stemness. The results showed that Ivosidenib treatment significantly reduced the number and volume of tumor spheres in SKOV3, CAOV3, OVCAR4, SK-3rd, and SKDDP cells [Figure 5D]. These data indicate that Ivosidenib reduces the stemness of ovarian cancer cells.

### Ivosidenib increases the sensitivity of ovarian cancer organoids to cisplatin

**Figure 6:**
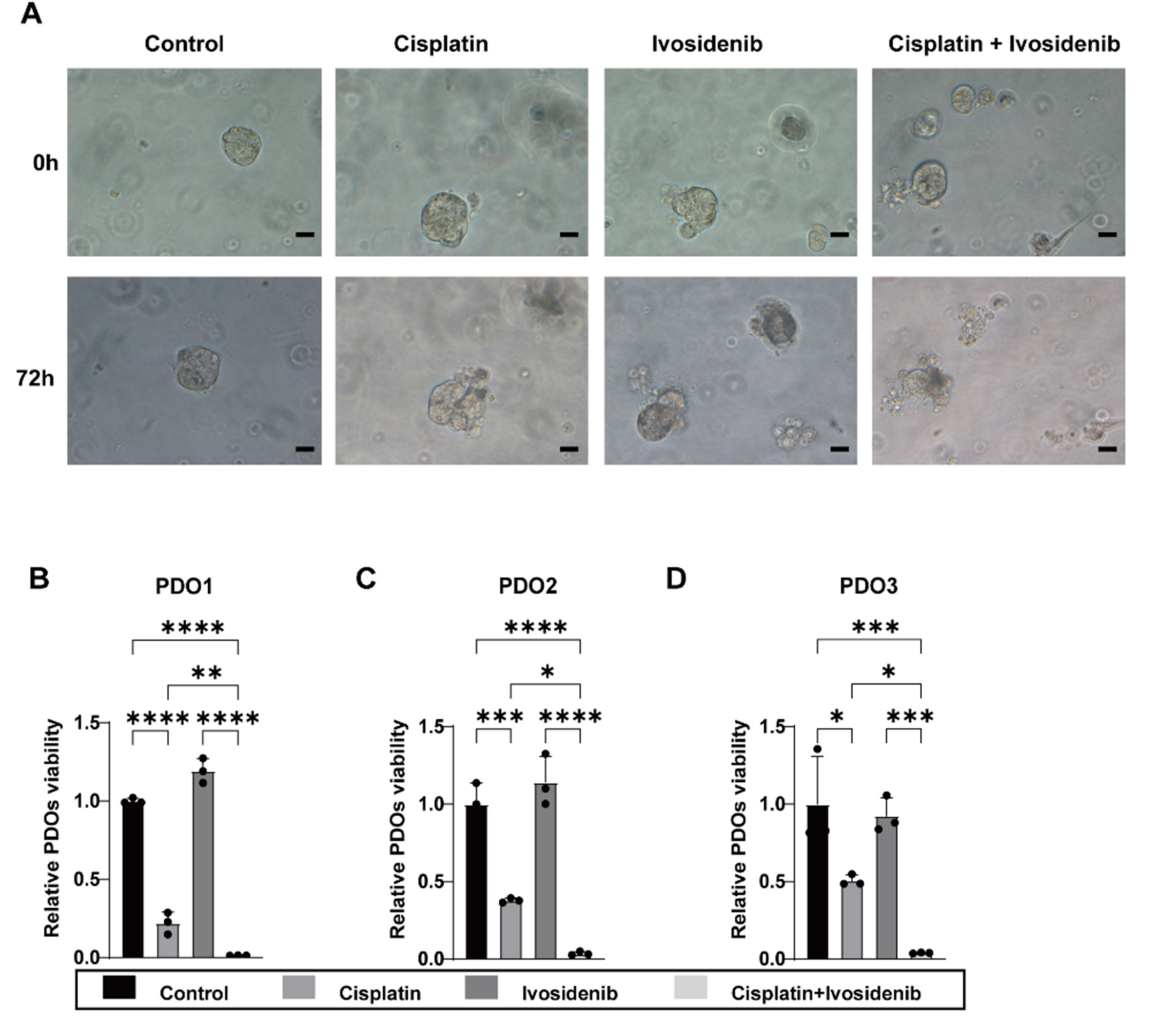
Ivosidenib increases the sensitivity of ovarian cancer organoids to cisplatin. (A) Representative images of PDO1 from ovarian cancer patient treated with DMSO, 10 μM Ivosidenib, 20 μM Cisplatin, or their combination for 72 h. Scale bar: 50 μm. (B-D) The CellTiter-Glo Luminescence Assay of PDO1, PDO2, and PDO3. Data are represented as mean ± SD. ns, not significant, *P <0.05; **P < 0.01; ***P < 0.001; ****P < 0.0001 using a one-way ANOVA followed by Tukey's post hoc test for multiple comparisons.

Patient-derived tumor organoid (PDO) models are widely recognized as important preclinical models for assessing drug response and efficacy^[21,22]^. To evaluate the feasibility of cisplatin combined with Ivosidenib in clinical treatment, we established three personalized PDO models derived from ovarian cancer patients. These PDO models were treated with cisplatin, Ivosidenib, and a combination of both drugs. The results demonstrated that compared to the control group, Ivosidenib alone had minimal cytotoxic effects on the PDO models, while cisplatin monotherapy exhibited moderate cytotoxic effects. Notably, the combination of cisplatin and Ivosidenib demonstrated significant cytotoxic effects on PDO models [Figure 6A]. In addition, the activity of PDO was quantified in the different treatment groups using CellTiter-Glo Luminescence Assays. Consistent with the *in vitro* observations, PDO activity remained unaffected by Ivosidenib alone, was partially reduced by cisplatin monotherapy, and was most significantly inhibited by the combination of Ivosidenib and cisplatin [Figure 6B-D].

## DISCUSSION

Chemoresistance remains a significant challenge in ovarian cancer treatment. In this context, the combination of platinum-based drugs with other therapeutic agents has emerged as a promising strategy to enhance efficacy and overcome chemoresistance^[23,24]^. In this study, we found that Ivosidenib alone did not exhibit significant cytotoxicity in ovarian cancer cells. However, Ivosidenib significantly enhanced the sensitivity of ovarian cancer cells to cisplatin. The effect exhibited a concentration-dependent trend when the concentration of Ivosidenib was lower than 10 μM. It reached a state of plateau when the concentration of Ivosidenib was over 10 μM. Additionally, Ivosidenib significantly attenuated the stem-like properties of ovarian cancer cells, partially reversing the cisplatin-induced enrichment of CSCs. Our findings suggest that Ivosidenib enhances cisplatin sensitivity in ovarian cancer cells, potentially through the suppression of CSC properties. This provides a theoretical basis for the potential clinical application of Ivosidenib in ovarian cancer treatment.

Ivosidenib, an inhibitor targeting mutant IDH1, is approved for treating IDH1-mutated malignancies, including AML and cholangiocarcinoma^[25,26]^. However, IDH1 mutations are relatively rare in ovarian cancer^[27]^, indicating that the cisplatin-sensitizing effects of Ivosidenib may involve non-canonical mechanisms. In the absence of mutant IDH1, it can also inhibit wild-type IDH1 activity^[16]^, which has been involved in the maintenance of CSC properties and chemoresistance^[28]^. Furthermore, cisplatin has been shown to induce the expression of CYP450 family members, increasing drug clearance. Ivosidenib can compete with cisplatin for CYP450 enzymes, leading to increased intracellular cisplatin concentrations and enhanced cisplatin sensitivity^[29-33]^. Nevertheless, the precise molecular mechanisms underlying these effects require further investigation.

Cisplatin and carboplatin behave similarly in terms of cytotoxicity. However, we did observe synergistic effects of Ivosidenib with cisplatin, but not with carboplatin. Although both drugs are platinum-based and kill cells by leading to intra- and inter-strand crosslinks of DNA and triggering cell apoptosis, cisplatin and carboplatin differ in molecular structure and pharmacological properties^[34]^, which may result in varied effects when they are combined with given drugs. For example, advanced glycation end products (AGEs) have been found to enhance the cytotoxicity of cisplatin, but not that of carboplatin. AGEs could reduce the expression of the multi-antimicrobial extrusion protein, a transporter implicated in the efflux of cisplatin, while it did not interact with carboplatin^[35]^. However, the mechanisms underlying the synergy of Ivosidenib and cisplatin are unexplored, which may provide some clues to explain the difference between cisplatin and carboplatin when they are combined with Ivosidenib.

To overcome the challenges posed by chemotherapy resistance in tumors, scientists continue to investigate new mechanisms of resistance and drug targets. However, the clinical application of new drugs requires further investigation into effective dosages and potential side effects. In this context, drug repurposing clinically approved drugs in novel therapeutic combinations can save significant time and costs, while the known safety profile facilitates faster development of treatment strategies. Recently, researchers discovered that stiripentol, a drug used for treating pediatric epilepsy, can act as a radiosensitizer in gastric cancer^[36]^. Additionally, drugs such as metformin and edaravone have been shown to target CSCs and reverse resistance in pancreatic cancer and brain tumors^[37,38]^. In our study, Ivosidenib was found to sensitize cisplatin, enhancing its therapeutic efficacy without escalating chemotherapy or radiotherapy doses, thus reducing cisplatin-associated side effects. Ivosidenib has been clinically available for several years, with a well-established safety profile that facilitates its clinical translation and enables earlier patient benefits.

In conclusion, while Ivosidenib alone has a limited effect on ovarian cancer, it markedly enhances cisplatin efficacy, reduces ovarian cancer stemness, and reverses platinum resistance. Moreover, the sensitizing effect of Ivosidenib on cisplatin has been validated in various cell models and PDO models. Therefore, Ivosidenib is a promising therapeutic agent, providing a novel treatment strategy for ovarian cancer patients, especially those with platinum resistance.

### Limitations of the study

While this study demonstrates that Ivosidenib sensitizes ovarian cancer cells to cisplatin and inhibits tumor stemness *in vitro*, several limitations should be acknowledged. First, the observed effects have not yet been validated *in vivo*, which limits the generalizability of our findings to clinical settings. To partially address this gap, we established PDO models as a complementary approach to better recapitulate the tumor microenvironment. Second, the molecular mechanisms underlying Ivosidenib-mediated cisplatin sensitization remain incompletely understood. Although we have proposed potential hypotheses, further mechanistic studies are required to validate these pathways and elucidate their roles in drug resistance and stemness regulation. Addressing these limitations will be a focus of our future research efforts.

## DECLARATIONS

### Availability of data and materials

All data are available on request from the authors.

### Copyright

© The Author(s) 2025.
