# Supplementary material for: Ivosidenib enhances cisplatin sensitivity in ovarian cancer by reducing cancer cell stemness
Source: Cancer Drug Resist. 2025 Apr 24;8:20. doi: 10.20517/cdr.2025.51 (PMC12059478; doi:10.20517/cdr.2025.51)
Supplement: Supplementary file 1 [file cdr-8-20-SupplementaryMaterials.pdf]

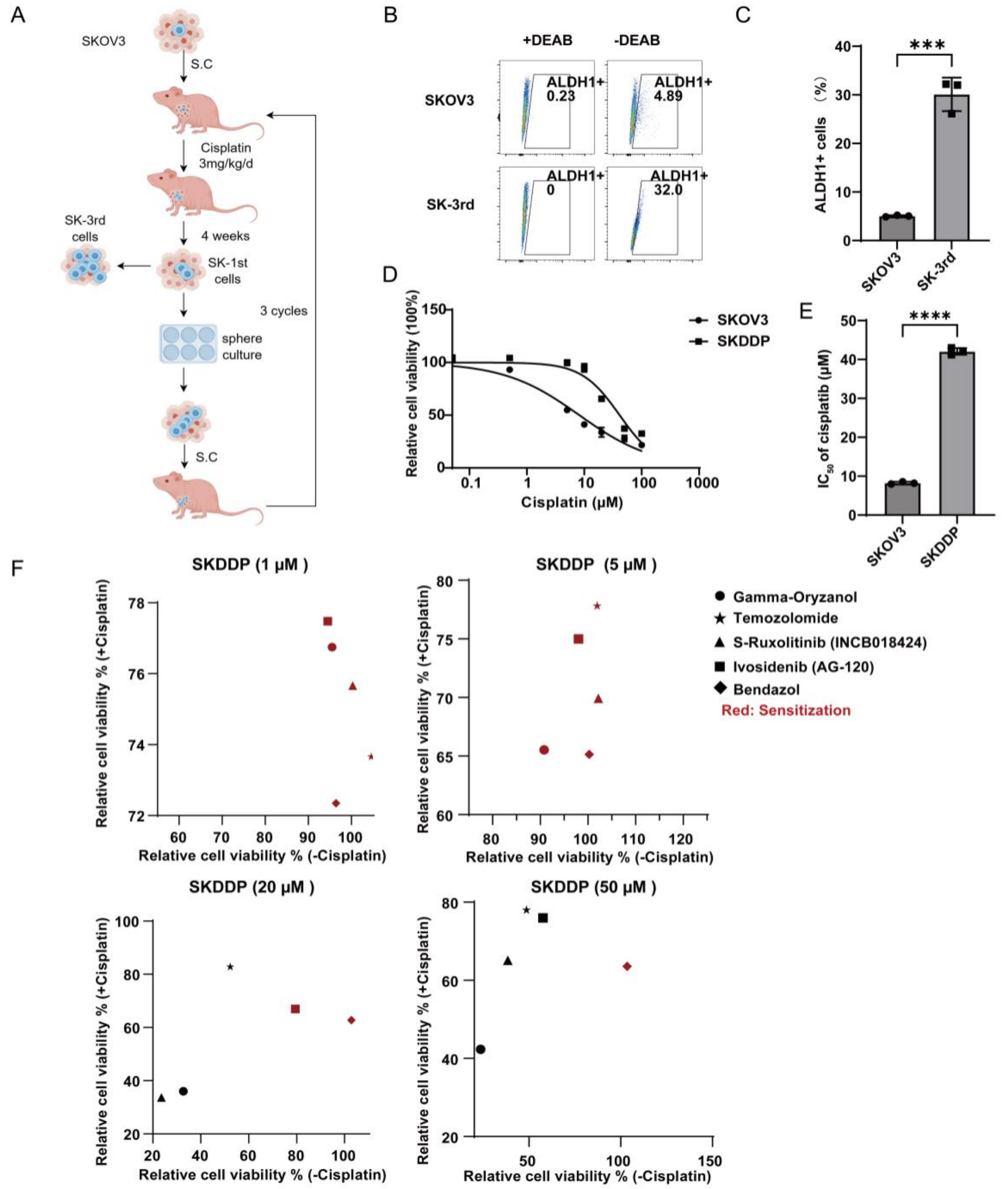

Supplementary Figure 1. Screening of a small molecule compound library shows that Ivosidenib enhances the sensitivity of ovarian cancer cells to cisplatin

(A) Schematic showing the method of SK-3rd model construction, as described in Materials and Methods.

(B) Representative FACS analysis showing the ALDH1+ cell populations in SK-3rd and SKOV3 cells.

(C) The proportion of ALDH1+ cells was analyzed statistically in SKOV3 and SK-3rd. Data is represented as mean ± SD of three biologically independent experiments. ****P* < 0.001 using an unpaired Student's t test.

(D)Dose-response curves of SKOV3 and SKDDP after cisplatin treatment for 72 hours detected by MTT assay. Data represent mean ± SD of three biologically independent experiments.

(E) The IC_{50} of the curves statistically in (D) were calculated by Graph Pad Prism 9.5.0. Data represent mean ± SD of three biologically independent experiments. *****P* < 0.0001 using an unpaired Student's t test.

(F) Scatter plot showing cell viability in SKDDP cells treated with the five selected compounds alone or

with the compounds plus cisplatin at a concentration of 1 μM, 5 μM, 20 μM, and 50 μM (n=4). The red dots represent drugs that have a sensitizing effect at that concentration.

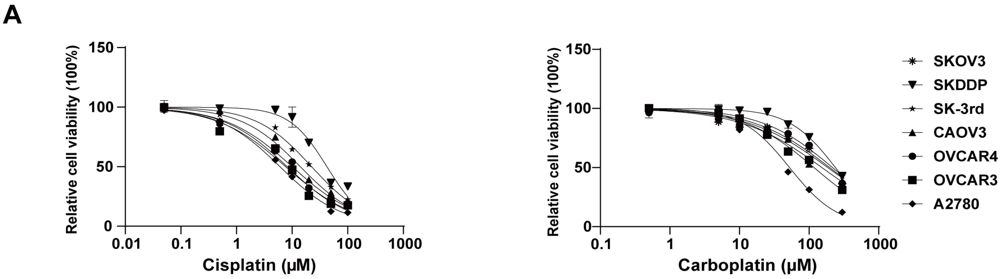

B

IC$_{50}$ (μM)	SKOV3	SKDDP	SK-3rd	CAOV3	OVCAR4	OVCAR3	A2780
Cisplatin	8.11 ± 0.06	41.94 ± 1.80	23.08 ± 0.59	13.97 ± 0.24	10.46 ± 0.49	7.61 ± 0.17	6.01 ± 0.04
Carboplatin	180.30 ± 4.04	239.6 ± 1.19	216.30 ± 4.92	156.87 ± 3.70	196.40 ± 1.98	117.13 ± 1.61	51.54 ± 0.72

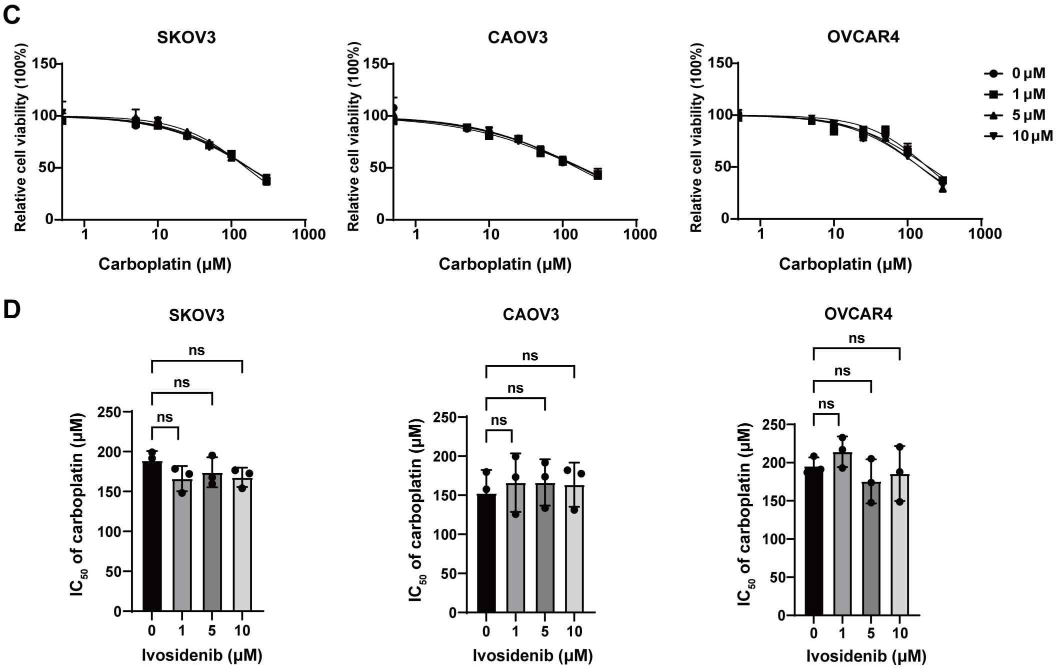

Supplementary Figure 2. Ivosidenib increases the sensitivity of ovarian cancer cells to cisplatin

(A)Dose-response curves of SKOV3, SKDDP, SK-3rd, CAOV3, OVCAR4, OVCAR3, and A2780 detected by MTT assay treated with cisplatin or carboplatin for 72 h.

(B)The IC$_{50}$ of SKOV3, SKDDP, SK-3rd, CAOV3, OVCAR4, OVCAR3, and A2780 to ciaplatin and carboplatin in (A) calculated by Graph Pad Prism 9.5.0. Data represent mean ± SD of three biologically independent experiments.

(C) Dose-response curves of SKOV3, CAOV3, and OVCAR4 detected by MTT assay treated with carboplatin and 0μM, 1μM, 5μM, and 10μM Ivosidenib for 72 h. Data represent mean ± SD of three biologically independent experiments.

(D) The IC$_{50}$ of SKOV3, CAOV3, and OVCAR4 to carboplatin in (C) calculated by Graph Pad Prism 9.5.0. Data represent mean ± SD of three biologically independent experiments. ns, not significant using a

one-way ANOVA followed by Tukey's post hoc test for multiple comparisons.

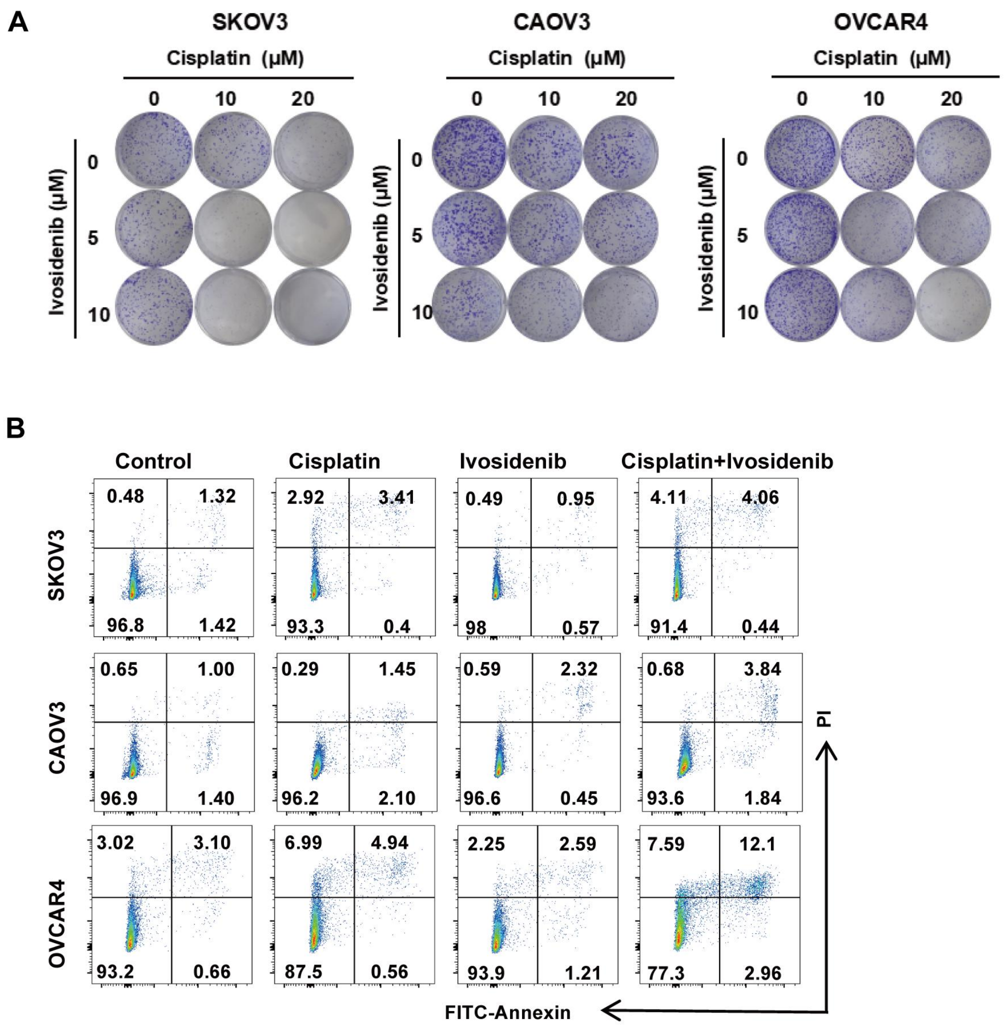

Supplementary Figure 3. Ivosidenib combined with cisplatin reduces colony formation and enhances apoptosis in ovarian cancer cells

(A) Representative images of colony formation assay for SKOV3, CAOV3, and OVCAR4 cells treated with Ivosidenib, cisplatin, or their combination as indicated.

(B) Representative images of cell apoptosis analysis in SKOV3, CAOV3, and OVCAR4 cells treated with 10 μM Ivosidenib, 10 μM cisplatin, or their combination for 72 h. Annexin V-positive cells were analyzed by flow cytometry after treatment.

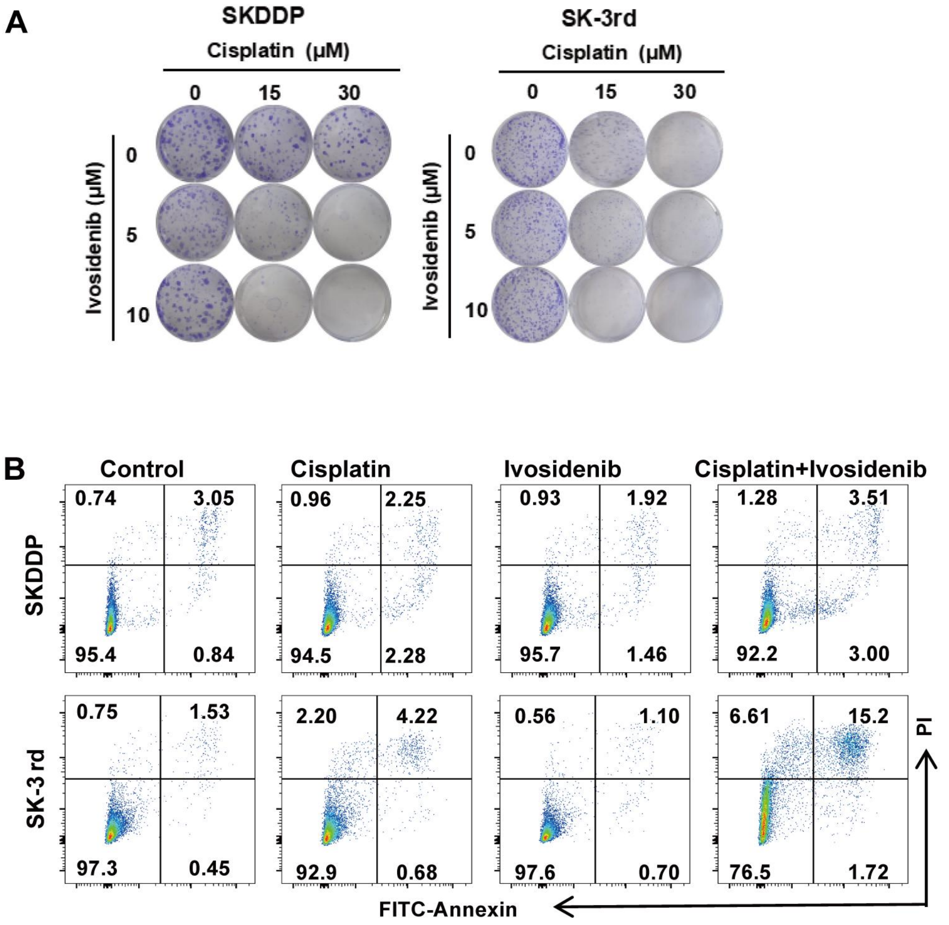

Supplementary Figure 4. Ivosidenib increases the sensitivity of SKDDP and SK-3rd to cisplatin

(A) Representative images of colony formation assay for SKDDP and SK-3rd cells treated with Ivosidenib, cisplatin, or their combination as indicated.

(B) Representative images of cell apoptosis analysis in SKDDP and SK-3rd cells treated with DMSO, 10 μM Ivosidenib, 10 μM cisplatin, or their combination for 72 h. Annexin V-positive cells were analyzed by flow cytometry after treatment.

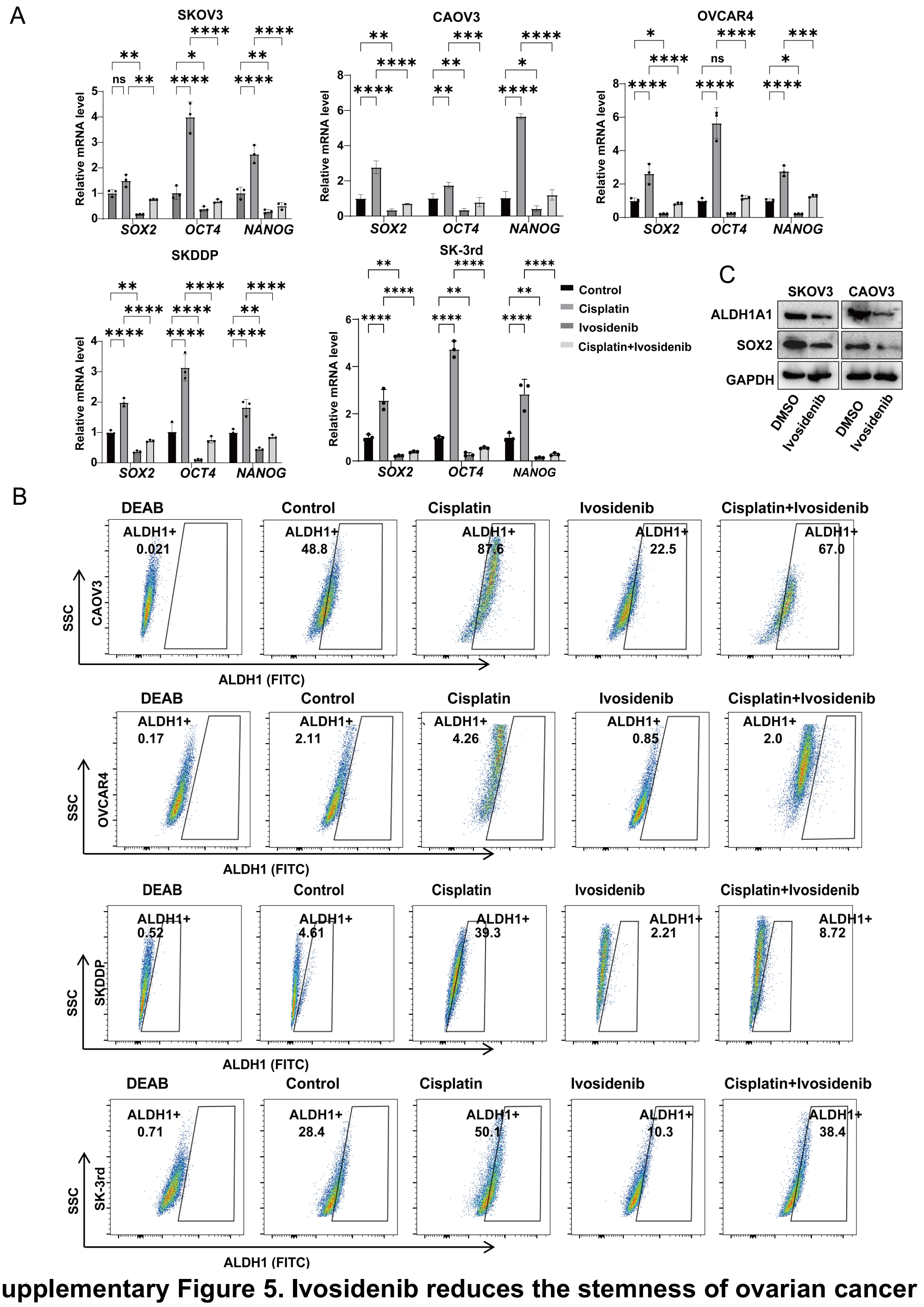

Supplementary Figure 5. Ivosidenib reduces the stemness of ovarian cancer cells

(A)Quantitative analysis of the mRNA levels of stemness-related genes, including *SOX2*, *OCT4*, and *NANOG* in SKOV3, CAOV3, OVCAR4, SKDDP, and SK-3rd treated with DMSO, 10 μM Ivosidenib, 10 μM cisplatin, or their combination for 72 h by qRT-PCR. Data is represented as mean ± SD of three biologically independent experiments. ns, not significant, **P* <0.05; ***P* < 0.01; ****P* < 0.001; *****P* < 0.0001 using a one-way ANOVA followed by Tukey's post hoc test for multiple comparisons.

(B)Representative images of ALDH1+ cells were analyzed using flow cytometry in CAOV3, OVCAR4, SKDDP, and SK-3rd cells treated with DMSO, 10 μM Ivosidenib, 10 μM cisplatin, or their combination for 72 h. DEAB was used as the negative control for ALDH1 activity.

(C) Western blot analysis of stemness markers ALDH1A1 and SOX2 in SKOV3 and CAOV3 cells treated with DMSO or 10 μM Ivosidenib.

Table S1. A collection of 105 compounds

Cat	Name	Rack Nun	Formulat	Worhing	Target	Pathway	Informati	M.w.	CAS Num	DMSO (m	DMSO (m	Water (m	Water (m	URL	Formula	Form	Synonym	SMILES	ALogP	HBA_Cou	HBD_Cou	Rotatable
S1007	Roxadust	L2000-01	in 10mM	10 μM	HIF	Angiogen	Roxadust	352.34	808118-4	70.00	198.67	<1		http://sel	C19H16N	free base	ASP1517	CC1=NC(=	2.306	4	2	5
S1030	Panobino	L2000-01	in 10mM	10 μM	HDAC	Epigeneti	Panobino	349.43	404950-8	69.00	197.46	<1		http://sel	C21H23N	Free Base	NVP-LBH!	CC1=C(CC	3.194	1	3	7
S1033	Nilotinib (	L2000-01	in 10mM	10 μM	Bcr-Abl	Angiogen	Nilotinib (	529.52	641571-1	27.00	50.99	<1		http://sel	C28H22F3	free base	N/A	CC1=C[N]	5.084	5	2	7
S1047	Vorinosta	L2000-01	in 10mM	10 μM	Autophag	Epigeneti	Vorinosta	264.30	149647-7	52.00	196.75	<1		http://sel	C14H20N	free base	SAHA, MK	ONC(=O)(	2.005	2	2	8
S1053	Entinosta	L2000-01	in 10mM	10 μM	HDAC	Epigeneti	Entinosta	376.41	209783-8	75.00	199.25	<1		http://sel	C21H20N	free base	SNDX-275	NC1=CC=(	2.071	4	3	7
S1055	Enzastaur	L2000-01	in 10mM	10 μM	PKC	TGF-beta,	Enzastaur	515.61	170364-5	30.00	58.18	<1		http://sel	C32H29N	free base	N/A	C[N]1C=C	4.320	3	1	5
S1085	Belinostat	L2000-01	in 10mM	10 μM	HDAC	Epigeneti	Belinostat	318.35	414864-0	64.00	201.04	<1		http://sel	C15H14N	free base	NSC72663	ONC(=O)/	1.800	3	2	5
S1168	Valproic a	L2000-01	in 10mM	10 μM	GABA Rec	Neuronal	Valproic a	166.19	1069-66-5	33.00	198.57	33.00	198.57	http://sel	C8H15Na	Sodium sa	N/A	CCCC(CCC	1.275	0	0	5
S1200	Decitabin	L2000-01	in 10mM	10 μM	DNA Met	Epigeneti	Decitabin	228.21	2353-33-5	45.00	197.19	10.00	43.82	http://sel	C8H12N4	free base	Deoxycyti	NC1=NC(=	-1.093	4	3	2
S1209	Fluoroura	L2000-01	in 10mM	10 μM	DNA/RNA	DNA Dam	Fluoroura	130.08	51-21-8	26.00	199.88	<1		http://sel	C4H3FN2	free base	NSC 1989	FC1=CNC(	-1.102	2	2	0
S1233	2-Methox	L2000-01	in 10mM	10 μM	HIF	Angiogen	2-Methox	302.41	362-07-2	60.00	198.41	<1		http://sel	C19H26O	free base	NSC 6598	COC1=C(C	3.821	1	2	1
S1237	Temozolc	L2000-01	in 10mM	10 μM	DNA/RNA	DNA Dam	Temozolc	194.15	85622-93	38.00	195.72	<1		http://sel	C6H6N6O	free base	CCRG810	CN1N=NC	0.052	5	1	1
S1373	Daptomy	L2000-01	in 10mM	10 μM	Anti-infec	DNA Dam	Daptomy	1620.67	103060-5	100.00	61.70	100.00	61.70	http://sel	C72H101	free base	LY146032	CCCCCCC(	-7.895	21	18	35
S1378	Ruxolitini	L2000-01	in 10mM	10 μM	JAK	JAK/STAT	Ruxolitini	306.37	941678-4	61.00	199.11	<1		http://sel	C17H18N	free base	N/A	N#CCC(C1	2.880	3	1	4
S1396	Resveratr	L2000-01	in 10mM	10 μM	Autophag	Autophag	Resveratr	228.24	501-36-0	45.00	197.16	<1		http://sel	C14H12O	free base	N/A	OC1=CC=(	3.090	0	3	2
S1421	Staurospc	L2000-02	in 10mM	10 μM	PKC,ADC	TGF-beta,	Staurospc	466.53	62996-74	93.00	199.34	<1		https://w	C28H26N	Free Base	CGP 4125	CNC1CC2(	3.819	3	2	2
S1573	Fasudil (H	L2000-01	in 10mM	10 μM	Autophag	Cell Cycle	Fasudil(H	327.83	105628-0	5.00	15.25	65.00	198.27	http://sel	C14H18Cl	Hydrochl	N/A	Cl.O=[S](=	0.570	3	1	2
S1703	Divalproe	L2000-01	in 10mM	10 μM	HDAC	Epigeneti	Divalproe	310.41	76584-70	62.00	199.74	62.00	199.74	http://sel	C16H31N	Sodium S	N/A	[Na+].CCC	4.025	1	0	10
S1756	Enoxacin	L2000-01	in 10mM	10 μM	Topoisom	DNA Dam	Enoxacin	320.32	74011-58	32.00	99.90	<1		http://sel	C15H17F	free base	AT-2266,	CCN1C=C	-1.483	3	1	3
S1782	Azacitidin	L2000-01	in 10mM	10 μM	DNA Met	DNA Dam	Azacitidin	244.20	320-67-2	48.00	196.56	<1		http://sel	C8H12N4	free base	NSC 1028	NC1=NC(=	-2.505	4	4	2
S1831	Carvedilo	L2000-01	in 10mM	10 μM	Adrenerg	Neuronal	Carvedilo	406.47	72956-09	81.00	199.28	<1		http://sel	C24H26N	free base	BM-1419(	COC1=CC	4.014	3	3	10
S1899	Nicotinan	L2000-01	in 10mM	10 μM	Sirtuin	DNA Dam	Nicotinan	122.12	98-92-0	24.00	196.53	24.00	196.53	http://sel	C6H6N2O	free base	Niacinam	NC(=O)C1	-0.319	2	1	1
S2105	Pantopra	L2000-01	in 10mM	10 μM	Proton Pu	Transmer	Pantopra	383.37	102625-7	76.00	198.24	<1		http://sel	C16H15F2	free base	N/A	COC1=C(C	2.737	6	1	7
S2250	(-)-Epigall	L2000-01	in 10mM	10 μM	DNA Met	DNA Dam	(-)-Epigall	458.37	989-51-5	72.00	157.08	23.00	50.18	http://sel	C22H18O	free base	EGCG	OC1=CC2	3.097	3	8	4
S2271	Berberine	L2000-01	in 10mM	10 μM	Anti-infec	Microbiol	Berberine	371.81	633-65-8	40.00	107.58	<1		http://sel	C20H18Cl	chloride	N/A	[Cl-].COC1	1.289	4	0	2
S2280	Chloroger	L2000-01	in 10mM	10 μM	Others	Others	Chloroger	354.31	327-97-9	71.00	200.39	18.00	50.80	http://sel	C16H18O	free base	NSC 4072	OC1CC(O)	-0.340	3	4	5
S2341	(-)-Parthe	L2000-01	in 10mM	10 μM	HDAC,NF-	NF-κB	(-)-Parthe	248.32	20554-84	49.00	197.33	<1		http://sel	C15H20O	free base	N/A	CC/1=C\C	2.923	3	0	0
S2347	Quercetir	L2000-01	in 10mM	10 μM	Others	Others	Quercetir	338.27	6151-25-3	67.00	198.07	<1		http://sel	C15H14O	Dihydrate	Sophoreti	O.O.OC1=	1.216	2	5	1
S2391	Quercetir	L2000-01	in 10mM	10 μM	Src,Sirtuir	Epigeneti	Quercetir	302.24	117-39-5	61.00	201.83	<1		http://sel	C15H10O	Free Base	Sophoreti	OC1=CC(=	1.630	2	5	1
S2410	Paeoniflo	L2000-01	in 10mM	10 μM	Others	Others	Paeoniflo	480.46	23180-57	96.00	199.81	96.00	199.81	http://sel	C23H28O	free base	N/A	CC12CC3(	-1.283	6	4	7
S2468	Fenbenda	L2000-01	in 10mM	10 μM	Anti-infec	Microbiol	Fenbenda	299.35	43210-67	4.00	13.36	<1		http://sel	C15H13N	free base	N/A	COC(=O)N	3.769	3	2	4
S2485	Mitoxantı	L2000-01	in 10mM	10 μM	Topoisom	DNA Dam	Mitoxantı	517.40	70476-82	89.00	172.01	89.00	172.01	http://sel	C22H30Cl	Dihydrocl	NSC-3017	Cl.Cl.OCCl	0.832	2	8	12
S2542	Phenform	L2000-01	in 10mM	10 μM	AMPK	PI3K/Akt/	Phenform	241.72	834-28-6	48.00	198.58	48.00	198.58	http://sel	C10H16Cl	Hydrochl	N/A	Cl.NC(=N)	1.339	0	3	6
S2554	Daphnetii	L2000-01	in 10mM	10 μM	PKA,EGFR	Angiogen	Daphnetii	178.14	486-35-1	35.00	196.47	<1		http://sel	C9H6O4	free base	N/A	OC1=CC=(	1.415	2	2	0
S2680	Ibrutinib (	L2000-01	in 10mM	10 μM	BTK	Angiogen	Ibrutinib (	440.50	936563-9	88.00	199.77	<1		http://sel	C25H24N	free base	N/A	NC1=C2C(	3.985	5	1	5
S2736	Fedratinib	L2000-01	in 10mM	10 μM	JAK	JAK/STAT	Fedratinib	524.68	936091-2	100.00	190.59	<1		http://sel	C27H36N	free base	N/A	CC1=C(NC	5.123	5	3	11
S2789	Tofacitini	L2000-01	in 10mM	10 μM	JAK	JAK/STAT	Tofacitini	312.37	477600-7	62.00	198.48	<1		http://sel	C16H20N	free base	N/A	CC1CCN(	1.512	3	1	3
S2851	Baricitinib	L2000-01	in 10mM	10 μM	JAK	JAK/STAT	Baricitinib	371.42	1187594-	74.00	199.24	<1		http://sel	C16H17N	free base	N/A	CC[S](=O)	0.360	5	1	5
S2902	S-Ruxoliti	L2000-01	in 10mM	10 μM	JAK	JAK/STAT	S-Ruxoliti	306.37	941685-3	61.00	199.11	5.00	16.32	http://sel	C17H18N	free base	N/A	N#CCC(C1	2.880	3	1	4
S3001	Clevudine	L2000-01	in 10mM	10 μM	DNA/RNA	DNA Dam	Clevudine	260.22	163252-3	52.00	199.83	52.00	199.83	http://sel	C10H13F	free base	N/A	CC1=CN(	-1.065	3	3	2
S3023	Bufexama	L2000-01	in 10mM	10 μM	COX	Neuronal	Bufexama	223.27	2438-72-4	45.00	201.55	<1		http://sel	C12H17N	free base	N/A	CCCCOC1	2.191	2	1	6
S3147	Entacapo	L2000-01	in 10mM	10 μM	Histone M	Epigeneti	Entacapo	305.29	130929-5	61.00	199.81	<1		http://sel	C14H15N	free base	OR-611	CCN(CC)C	1.658	1	2	5
S3273	Hypericin	L2000-02	in 10mM	10 μM	MAO	Metabolis	Hypericin	504.44	548-04-9	100.00	198.24	-1.00		http://wv	C30H16O	free base	Hyp, HY	CC1=C2C3	5.040	2	6	0
S3341	Palmitole	L2000-02	in 10mM	10 μM	AMPK	PI3K/Akt/	Palmitole	254.41	373-49-9	liquid, which can be dissolved in any pro				https://w	C16H30O	Free Base	POA, Paln	CCCCCC\(	5.948	1	0	13
S3589	Bendazol	L2000-02	in 10mM	10 μM	NOS	Immunolc	Bendazol	208.26	621-72-7	42.00	201.67	<1		http://wv	C14H12N	Free Base	2-Benzylb	C(C1=NC2	3.305	1	1	2
S3592	Benzenet	L2000-02	in 10mM	10 μM	HDAC	DNA Dam	4-Phenylb	164.20	1821-12-1	33.00	200.97	<1		http://wv	C10H12O	Free Base	Benzenet	OC(=O)CC	2.407	1	0	4
S3609	Berbamin	L2000-01	in 10mM	10 μM	Bcr-Abl	Angiogen	Berbamin	681.65	6078-17-7	100.00	146.70	100.00	146.70	http://sel	C37H42Cl	dihydroch	N/A	Cl.Cl.COC	7.756	5	1	3
S3639	Tacrine h	L2000-01	in 10mM	10 μM	AChR	Neuronal	Tacrine is	198.26	206658-9	50.00	252.19	50.00	252.19	http://sel	C13H14N	hydrochlc	Tacrine, T	NC1=C2C	4.258	1	1	0
S3694	Glucosam	L2000-01	in 10mM	10 μM	Others	Others	Glucosam	215.63	66-84-2	4.00	18.55	43.00	199.42	http://sel	C6H14ClN	hydrochlc	2-Amino-	Cl.NC1C(	-2.422	1	4	1
S3781	Ginkgolid	L2000-01	in 10mM	10 μM	Others	Others	Ginkgolid	440.40	15291-76	88.00	199.82			http://sel	C20H24O	free base	BN-52022	CC1C(=O)	-1.753	7	3	1
S3850	Glucosam	L2000-01	in 10mM	10 μM	Others	Others	Glucosam	277.25	29031-19	4.00	14.43	55.00	198.38	http://sel	C6H15NO	sulfate	D-Glucosa	NC(C=O)C	-3.688	1	5	5
S3944	Valproic a	L2000-01	in 10mM	10 μM	HDAC	Epigeneti	Valproic a	144.21	99-66-1	liquid, which can be dissolved in any pro				http://sel	C8H16O2	free base	2-Propylv	CCCC(CCC	2.749	1	0	5
S3957	Gamma-C	L2000-01	in 10mM	10 μM	Others	Others	Gamma-c	602.89	11042-64	100.00	165.87			http://sel	C40H58O	free base	N/A	COC1=CC	9.806	3	1	9
S3984	Nordihyd	L2000-01	in 10mM	10 μM	Lipoxyger	Metabolis	Nordihyd	302.36	500-38-9	60.00	198.44	<1		http://sel	C18H22O	free base	N/A	CC(CC1=C	4.708	0	4	5
S4021	Tolcapon	L2000-01	in 10mM	10 μM	Transfera	Metabolis	Tolcapon	273.24	134308-1	55.00	201.29	<1		http://sel	C14H11N	free base	Ro 40-75	CC1=CC=(	3.130	1	2	3
S4023	Procaine	L2000-01	in 10mM	10 μM	NMDAR,S	Transmer	Procaine	272.77	51-05-8	55.00	201.64	55.00	201.64	http://sel	C13H21Cl	HCl	Novocain	Cl.CCN(CC	2.157	2	1	7
S4125	Sodium P	L2000-01	in 10mM	10 μM	HDAC	DNA Dam	Sodium p	186.18	1716-12-7	8.00	42.97	30.00	161.13	http://sel	C10H11N	Sodium S	N/A	[Na]OC(=(	2.497	2	0	5
S4246	Tranylcyp	L2000-01	in 10mM	10 μM	MAO	Metabolis	Tranylcyp	169.65	1986-47-6	33.00	194.52	33.00	194.52	http://sel	C9H12ClN	Hydrochl	SKF-385 H	Cl.NC1CC	2.309	0	1	1
S4268	Flufenam	L2000-01	in 10mM	10 μM	Immunolc	Immunolc	Flufenam	281.23	530-78-9	56.00	199.13	<1		http://sel	C14H10F3	free base	N/A	OC(=O)C1	3.952	1	1	4
S4294	Procainar	L2000-01	in 10mM	10 μM	DNA Met	Transmer	Procainar	271.79	614-39-1	54.00	198.68	54.00	198.68	http://sel	C13H22Cl	Hydrochl	N/A	Cl.CCN(CC	1.509	1	2	6
S4295	Meclofen	L2000-01	in 10mM	10 μM	COX	Neuronal	Meclofen	318.13	6385-02-(	63.00	198.03	63.00	198.03	http://sel	C14H10Cl	Sodium S	N/A	CC1=C(Cl)	4.915	2	1	4
S4357	Tacrine H	L2000-01	in 10mM	10 μM	Others	Others	Tacrine is	234.72	1684-40-8	46.00	195.98	46.00	195.98	http://wv	C13H15Cl	hydrochlc	N/A	Cl.NC1=C	3.172	1	1	0
S4538	Pantopra	L2000-01	in 10mM	10 μM	Proton Pu	Transmer	Pantopra	405.35	138786-6	81.00	199.83	81.00	199.83	http://sel	C16H14F2	Free Base	SKF96022	[Na+].COC	2.883	6	0	7
S4561	Danthron	L2000-01	in 10mM	10 μM	Others	Others	Danthron	240.21	117-10-2	4.00	16.65	<1		http://sel	C14H8O4	free base	chrysazin	OC1=CC=(	2.324	2	2	0
S4589	Amodiaqu	L2000-01	in 10mM	10 μM	Transfera	Epigeneti	Amodiaqu	464.81	6398-98-7	92.00	197.93	29.00	62.39	http://sel	C20H28Cl	dihydroch	N/A	O.O.Cl.Cl.	5.109	1	2	6
S4609	Diflunisal	L2000-01	in 10mM	10 μM	COX	Neuronal	Diflunisal	250.20	22494-42	50.00	199.84	<1		http://sel	C13H8F2(	free base	Dolobid, I	OC(=O)C1	3.147	1	1	2
S4635	Cyprohep	L2000-01	in 10mM	10 μM	5-HT Rece	Neuronal	Cyprohep	350.88	41354-29	70.00	199.50	<1		http://sel	C21H22Cl	Hydrochl	Periactin	Cl.CN1CC(	5.478	0	0	0
S4668	Procaine	L2000-01	in 10mM	10 μM	Sodium C	Transmer	Procaine	236.31	59-46-1	47.00	198.89	<1		http://sel	C13H20N	Free Base	Procaine,	CCN(CC)C	1.775	2	1	7
S4709	Latanoprc	L2000-01	in 10mM	10 μM	Immunolc	Immunolc	Latanoprc	432.59	130209-8	liquid, which can be dissolved in any pro				http://sel	C26H40O	Free Base	Xalatan, F	CC(C)OC(=	4.365	2	3	14
S4735	Salvianoli	L2000-01	in 10mM	10 μM	Sirtuin	Epigeneti	Salvianoli	718.61	121521-9	100.00	139.16	100.00	139.16	http://sel	C36H30O	free base	Lithosper	OC(=O)C(	4.718	7	7	14
S4895	Nilotinib I	L2000-02	in 10mM	10 μM	Bcr-Abl	Angiogen	Nilotinib (	583.99	923288-9	100.00	171.24	<1		http://wv	C28H25Cl	Hydrochl	Tasigna, A	O.Cl.CC1=	5.259	5	2	7
S4929	Enasideni	L2000-02	in 10mM	10 μM	Dehydrog	Metabolis	Enasideni	569.48	1650550-	100.00	175.60	<1		http://wv	C20H21F6	Mesylate	Idhifa, AG	CC(C)(O)C	4.464	5	2	8
S5001	Tofacitini	L2000-01	in 10mM	10 μM	JAK	JAK/STAT	Tofacitini	504.49	540737-2	100.00	198.22	<1		http://sel	C22H28N	Citrate	N/A	CC1CCN(	0.193	6	1	8
S5020	Tilorone (	L2000-01	in 10mM	10 μM	Anti-infec	Microbiol	Tilorone (	483.47	27591-69	43.00	88.94	96.00	198.56	http://sel	C25H36Cl	dihydroch	N/A	Cl.Cl.CCN(	5.326	3	0	12
S5205	Nilotinib I	L2000-01	in 10mM	10 μM	Bcr-Abl	Angiogen	Nilotinib I	565.98	923288-9	100.00	176.69	-1.00	-1.77	http://sel	C28H23Cl	hydrochlc	AMN-107	Cl.CC1=C[	5.466	5	2	7
S5209	Enoxacin	L2000-01	in 10mM	10 μM	Anti-infec	Microbiol	Enoxacin	347.34	84294-96	4.00	11.52			http://sel	C15H17F	sesquihyc	Enoxacin	CCN1C=C	-0.749	3	1	3
S5243	Ruxolitini	L2000-01	in 10mM	10 μM	JAK	JAK/STAT	Ruxolitini	404.36	1092939-	80.00	197.84	29.00	71.72	http://sel	C17H21N	phosphat	INCB0184	O[P](O)(O	2.412	3	1	4
S5284	Adenosin	L2000-01	in 10mM	10 μM	AMPK	PI3K/Akt/	Adenosin	365.24	18422-05	9.00	24.64			http://sel	C10H16N	hydrate	5'-Adenyl	O.NC1=N(	-2.160	5	3	4
S5440	Berberine	L2000-01	in 10mM	10 μM	Anti-infec	Microbiol	Berberine	433.43	633-66-9	20.00	46.14			http://sel	C20H19N	sulfate	Umbellati	COC1=C(C	1.111	4	0	2
S5457	Curculigo	L2000-01	in 10mM	10 μM	Immunolc	Immunolc	Curculigo	466.44	85643-19	93.00	199.38	15.00	32.16	http://sel	C22H26O	free base	Curculigo	COC1=C(C	0.822	6	5	9
S5458	Verbasco	L2000-01	in 10mM	10 μM	Immunolc	Immunolc	Verbasco	624.59	61276-17	100.00	160.11	100.00	160.11	http://sel	C29H36O	free base	Acteoside	CC1OC(O(	0.484	6	9	11
S5500	Amodiaqu	L2000-01	in 10mM	10 μM	HnMT	Immunolc	Amodiaqu	428.78	69-44-3	56.00	130.60			http://sel	C20H24Cl	dihydroch	N/A	Cl.Cl.CCN(	5.523	1	2	6
S5554	Lanatosid	L2000-01	in 10mM	10 μM	ATPase	Transmer	Lanatosid	985.12	17575-22	liquid, which can be dissolved in any pro				http://sel	C49H76O	free base	N/A	CC1OC(CC	0.631	12	7	12
S5582	Cytarabin	L2000-01	in 10mM	10 μM	DNA/RNA	DNA Dam	Cytarabin	279.68	69-74-9	55.00	196.65			http://sel	C9H14ClN	hydrochlc	N/A	Cl.NC1=N	-2.014	3	4	2
S5608	Pantopra	L2000-02	in 10mM	10 μM	ATPase	Transmer	Pantopra	864.75	164579-3	100.00	115.64	17.00	19.66	http://wv	C32H34F4	Sodium h	Protonix,	O.O.O.[Na	5.145	12	0	14
S5703	Carvedilo	L2000-01	in 10mM	10 μM	Others	Others	Carvedilo	513.48	610309-8	100.00	198.23	-1.00	-1.98	http://sel	C24H29N	free base		COC1=C(C	4.280	3	3	10
S5754	Baricitinib	L2000-02	in 10mM	10 μM	JAK	JAK/STAT	Baricitinib	469.41	1187595-	94.00	200.25	-1.00		http://wv	C16H20N	phosphat	INCB-028	CC[S](=O)	-0.108	5	1	5
S5948	Amodiaqu	L2000-02	in 10mM	10 μM	Anti-infec	Microbiol	Amodiaqu	355.86	86-42-0	71.00	199.52	<1		http://wv	C20H22Cl	Free Base	N/A	CCN(CC)C	4.760	1	2	6
S5958	Metformi	L2000-02	in 10mM	10 μM	AMPK	PI3K/Akt/	Metformi	129.16	657-24-9	26.00	201.30	26.00	201.30	http://wv	C4H11N5	Free Base	1, 1-Dime	CN(C)C(=I	-0.742	0	2	3
S5967	Berberine	L2000-02	in 10mM	10 μM	Anti-infec	Immunolc	Berberine	389.83	68030-18	78.00	200.09	39.00	100.04	http://wv	C20H20Cl	hydrate	Natural Y	O.[Cl-].CO	1.081	4	0	2
S6221	Methyl ci	L2000-02	in 10mM	10 μM	Others	Others	Methyl ci	162.19	103-26-4	32.44	200.00			http://wv	C10H10O	free base	N/A	COC(=O)/	2.153	2	0	3
S6400	Glucosam	L2000-02	in 10mM	10 μM	Others	Others	Glucosam	179.17	3416-24-8	23.00	128.37	36.00	200.93	http://wv	C6H13NO	Free Base	2-amino-	NC(C=O)C	-3.081	1	5	5
S7650	Peficitinib	L2000-02	in 10mM	10 μM	JAK	JAK/STAT	Peficitinib	326.39	944118-0	65.00	199.15	<1		http://sel	C18H22N	free base	N/A	NC(=O)C1	1.023	2	3	3
S7663	Ruboxista	L2000-02	in 10mM	10 μM	PKC	TGF-beta,	LY333531	505.01	169939-9	50.00	99.01	<1		http://sel	C28H29Cl	hydrochlc	Ruboxista	Cl.CN(C)C	3.861	3	1	2
S7953	ETC-1002	L2000-02	in 10mM	10 μM	AMPK,LD	PI3K/Akt/	ETC-1002	344.49	738606-4	68.00	197.39	<1		http://sel	C19H36O	free base	Bempedo	CC(C)(CCC	5.180	2	1	14
S8162	Upadaciti	L2000-02	in 10mM	10 μM	JAK	JAK/STAT	Upadaciti	380.37	1310726-	76.00	199.81	<1		http://wv	C17H19F3	free base	N/A	CCC1CN	1.967	3	2	4
S8195	Oclacitinil	L2000-02	in 10mM	10 μM	JAK	JAK/STAT	Oclacitinil	453.51	1640292-	90.00	198.45	18.00	39.69	http://sel	C19H27N	maleate	PF-03394	CN[S](=O)	1.758	6	2	7
S8205	Enasideni	L2000-02	in 10mM	10 μM	Dehydrog	Metabolis	Enasideni	473.38	1446502-	94.00	198.57	<1		http://sel	C19H17F6	free base	N/A	CC(C)(O)C	4.878	5	2	8
S8206	Ivosidenib	L2000-02	in 10mM	10 μM	Dehydrog	Metabolis	Ivosidenib	582.96	1448347-	100.00	171.54	<1		http://sel	C28H22Cl	free base		FC1=CN=(	3.004	5	1	7
S8567	Tucidinos	L2000-02	in 10mM	10 μM	HDAC	Epigeneti	Chidamid	390.41	1616493-	78.00	199.79	<1		http://sel	C22H19F	free base	HBI-8000,	NC1=C(N(	2.182	3	3	6
S9024	AKBA	L2000-02	in 10mM	10 μM	Cancer	HIF/HIF P	Acetyl-11	512.72	67416-61	100.00	195.04				C32H48O5		FA42758	CC1CCC2(	6.081	4	0	3
S9046	Berberine	L2000-02	in 10mM	10 μM	Others	Others	Berberine	336.36	2086-83-1	45.00	133.78				C20H18N	free base	5, 6-Dihyc	COC1=C(C	3.962	4	0	2
S9141	Berbamin	L2000-02	in 10mM	10 μM	Others	Others	Berbamin	608.72	478-61-5	100.00	164.28				C37H40N	free base		COC1=C2(	6.992	5	1	3
S9192	Vitexin	L2000-02	in 10mM	10 μM	Others	antioxida	An apiger	432.38	3681-93-4	47.00	108.70				C21H20O	free base		OCC1OC((	0.018	3	7	3
S9656	Enarodus	L2000-02	in 10mM	10 μM	HIF	Angiogen	Enarodus	340.33	1262132-	68.00	199.81	<1		https://w	C17H16N	Free Base	N/A	OC(=O)CN	1.511	4	2	6
